# Single-cell exome sequencing reveals polyclonal seeding and TRPS1 mutations in colon cancer metastasis

**DOI:** 10.1038/s41392-024-01960-8

**Published:** 2024-09-23

**Authors:** Jianqiang Cai, Weilong Zhang, Yalan Lu, Wenjie Liu, Haitao Zhou, Mei Liu, Xinyu Bi, Jianmei Liu, Jinghua Chen, Yanjiang Yin, Yiqiao Deng, Zhiwen Luo, Yi Yang, Qichen Chen, Xiao Chen, Zheng Xu, Yueyang Zhang, Chaoling Wu, Qizhao Long, Chunyuan Huang, Changjian Yan, Yan Liu, Lei Guo, Weihua Li, Pei Yuan, Yucheng Jiao, Wei Song, Xiaobing Wang, Zhen Huang, Jianming Ying, Hong Zhao

**Affiliations:** 1 Department of Hepatobiliary Surgery, State Key Laboratory of Molecular Oncology, National Cancer Center/National Clinical Research Center for Cancer/Cancer Hospital, Chinese Academy of Medical Sciences and Peking Union Medical College, Beijing, China; 2 Key Laboratory of Gene Editing Screening and R & D of Digestive System Tumor Drugs, Chinese Academy of Medical Sciences and Peking Union Medical College, Beijing, China; 3 Department of Hematology, Lymphoma Research Center, Peking University Third Hospital, Beijing, China; 4 Key Laboratory of Human Disease Comparative Medicine, Chinese Ministry of Health, Beijing Key Laboratory for Animal Models of Emerging and Remerging Infectious Diseases, Institute of Laboratory Animal Science, Chinese Academy of Medical Sciences and Comparative Medicine Center, Peking Union Medical College, Beijing, China; 5 Department of Biochemistry and Molecular Biology, State Key Laboratory of Medical Molecular Biology, Institute of Basic Medical Sciences Chinese Academy of Medical Sciences, School of Basic Medicine Peking Union Medical College, Beijing, China; 6 Department of Colorectal Surgery, State Key Laboratory of Molecular Oncology, National Cancer Center/National Clinical Research Center for Cancer/Cancer Hospital, Chinese Academy of Medical Sciences and Peking Union Medical College, Beijing, China; 7 State Key Laboratory of Molecular Oncology, National Cancer Center/National Clinical Research Center for Cancer/Cancer Hospital, Chinese Academy of Medical Sciences and Peking Union Medical College, Beijing, China; 8 Department of Pathology, State Key Laboratory of Molecular Oncology, National Cancer Center/National Clinical Research Center for Cancer/Cancer Hospital, Chinese Academy of Medical Sciences and Peking Union Medical College, Beijing, China

**Keywords:** Gastrointestinal cancer, Gene expression analysis, Genomic analysis

## Abstract

Liver metastasis remains the primary cause of mortality in patients with colon cancer. Identifying specific driver gene mutations that contribute to metastasis may offer viable therapeutic targets. To explore clonal evolution and genetic heterogeneity within the metastasis, we conducted single-cell exome sequencing on 150 single cells isolated from the primary tumor, liver metastasis, and lymphatic metastasis from a stage IV colon cancer patient. The genetic landscape of the tumor samples revealed that both lymphatic and liver metastases originated from the same region of the primary tumor. Notably, the liver metastasis was derived directly from the primary tumor, bypassing the lymph nodes. Comparative analysis of the sequencing data for individual cell pairs within different tumors demonstrated that the genetic heterogeneity of both liver and lymphatic metastases was also greater than that of the primary tumor. This finding indicates that liver and lymphatic metastases arose from clusters of circulating tumor cell (CTC) of a polyclonal origin, rather than from a single cell from the primary tumor. Single-cell transcriptome analysis suggested that higher EMT score and CNV scores were associated with more polyclonal metastasis. Additionally, a mutation in the *TRPS1* (Transcriptional repressor GATA binding 1) gene, TRPS1 R544Q, was enriched in the single cells from the liver metastasis. The mutation significantly increased CRC invasion and migration both in vitro and in vivo through the TRPS1^R544Q^/ZEB1 axis. Further TRPS1 mutations were detected in additional colon cancer cases, correlating with advanced-stage disease and inferior prognosis. These results reveal polyclonal seeding and *TRPS1* mutation as potential mechanisms driving the development of liver metastases in colon cancer.

## Introduction

Colon cancer ranks among the most prevalent and deadly cancers globally. It has been reported that about half of colon cancer patients die from hepatic failure and other syndromes associated with liver metastasis.^1^ The 5-year survival rate for metastatic colorectal cancer (mCRC) is below 15%. Liver metastasis often responds differently to therapy compared to the primary tumor or lymphatic metastasis. Although multiple studies have described the clonal evolution of colon cancer, there are conflicting opinions regarding the origin of metastasis. Several studies suggest that liver metastasis is derived from single circulating tumor cells (CTCs) from the primary tumor. Individual CTCs possesses the capacity to detach from the primary tumor site and disseminate to other tissues and organs via the bloodstream. After breaking away from the primary tumor, these single aggressive CTCs travel through the bloodstream to the liver, where they form new clonal populations. They are highly similar at the genomic level because they are derived from the same original cancer cell. Whereas other studies have found that CTC clusters exhibit a significantly greater potential to form metastasis. Compared to the relatively weak immune escape ability of individual CTC in the face of immune system surveillance and attack, cells in CTC clusters can support each other, improving survival, aggressiveness, and treatment resistance. Cells in CTC clusters may have different genomic characteristics that allow them to adapt and grow faster in new environments.^2–4^ In addition, recent studies show that the majority of liver metastases are derived directly from primary tumor rather than through lymphatic metastasis as previously recognized.^5^

Cancer metastasis is a complex process influenced by numerous factors. A hypoxic microenvironment can promote the reprogramming of tumor cell energy metabolism, enhancing their aggressiveness and metastasis, and rendering tumor cells resistant to chemotherapy drugs and radiotherapy. Hypoxia, on one hand, can induce the expression of specific genes involved in drug metabolism and DNA repair, allowing tumor cells to evade the cytotoxic effects of therapeutic agents. On the other hand, the hypoxic microenvironment can also reduce the penetration and distribution of drugs within tumor tissues, making it difficult for drugs to reach effective concentrations.^6^ The genes of tumor cells themselves also mutate, making the cells more aggressive and migratory. The abnormal activation of proto-oncogenes can lead to an excessive enhancement of cell proliferation signals, causing cells to lose normal growth regulation and resulting in unlimited proliferation. Simultaneously, the inactivation of tumor suppressor genes will cause cells to lose proliferation inhibition, further exacerbating abnormal cell proliferation. Epithelial-mesenchymal transition (EMT) is also a key driver of cancer metastasis.

As is well known, EMT is a process in which epithelial cells transdifferentiate into mesenchymal cells, enhancing their motility and developing an invasive phenotype in the CRC developmental stage.^7^ During the EMT process, epithelial markers like E-cadherin, ZO-1, and occludin are downregulated, while mesenchymal markers such as fibronectin, vimentin, and N-cadherin are upregulated. ZEB1 functions as a key transcription factor in EMT by directly binding to the promoter of the E-cadherin epithelial marker, leading to its suppression. Correspondingly, ZEB1 is positively correlated with mesenchymal phenotypes, the aggressiveness of carcinomas, and poor clinical survival.

Transcriptional repressor GATA binding 1 (TRPS1) is a vital regulator in EMT pathway. There is growing evidence suggesting a multifaceted role for TRPS1 in tumor biology. For example, a recent study demonstrated a replication-boosting role of TRPS1 in H3K9me3-marked heterochromatic origin activation and cancer genome evolution.^8^ Another study suggested that TRPS1 might act to enhance FOXA1 expression by engaging with its promoter, further broadening its previously understood role as a transcriptional repressor.^9^ Several other studies have reported that TRPS1 could act as a breast cancer driver, but the role of TRPS1 in colon cancer remains unclear. A prior study demonstrated that TRPS1 expression is elevated in colon cancer and that high TRPS1 levels are significantly correlated with positive lymph node metastasis and more advanced pathological stages in colon cancer patients.^10^ Information is scarce regarding the identification of TRPS1 mutations or the elucidation of TRPS1’s mechanism in colon cancer.

In this study, we sequenced the exomes of 150 single cells from primary tumor, liver metastasis, lymphatic metastasis, and an independent adenoma from a late-stage colon cancer patient. We compared the genomes of the primary tumor and its metastases at single-cell resolution and found that both liver and lymphatic metastases were derived directly from the primary tumor but with higher heterogeneity. This supports a model in which metastases may arise from tumor cell clusters of a polyclonal origin in the primary tumor. In addition, a mutation in the *TRPS1* gene, leading to TRPS1 R544Q, was enriched in the liver metastasis. TRPS1 R544Q mutant cells significantly accelerate cancer cell migration and invasion both in vitro and in vivo. RNA sequencing enriched EMT process, and ZEB1 was selected as a key downstream target. ChIP-qPCR and luciferase reporter assay showed mutant TRPS1 direct binding to ZEB1 promoter, and activating its transcription. Rescue assays confirmed the TRPS1 R544Q/ZEB1 axis for CRC metastasis. In total, TRPS1 R544Q promotes CRC metastasis, ultimately leading to a worse patient prognosis.

## Results

### Identification of somatic mutations with high specificity in a single colorectal cancer patient

We obtained tissue samples from a 76-year-old male with well-defined stage IV metastatic colon adenocarcinoma according to the WHO classification. In addition to sampling primary tumors with matched normal tissues, we also collected fresh tissue samples from lymphatic metastasis, liver metastasis, white blood cells, and an independent benign adenoma. To analyze the intra-tumor and inter-tumor heterogeneity at the single-cell level, we isolated single cells from four different sections of the primary tumor as well as from all the other tissues collected from this patient (Supplementary Table 1-2). Exome sequencing achieved at least a 30-fold depth on the target regions for each cell, with 83.8% of the regions covered at least one time (Supplementary Fig. 1).

For each of the tissues and single cells, we first used SOAPsnp to predict single nucleotide polymorphisms (SNPs). Highly confident homozygous sites from all tissue sequencing results were used to estimate the false-positive (FP) rate in the single-cell analysis. The average FP rate was 2.68e−06 (Supplementary Fig. 2), which was substantially lower than the sequencing error rate. In addition to FP mutations, the false-negative discovery of true mutations also has significant effects on heterogeneity and evolution analyses. However, the incidence of allele dropout (ADO), which is the random loss of amplification of one allele at a heterozygous locus in a single cell, is inevitable. To estimate the ADO rate, we selected highly confident germline heterozygous SNP sites as controls. For each single cell, confident homozygous SNP calls at these sites were considered ADO events. The median estimated ADO rate was 10.3% (Supplementary Fig. 2), which was slightly lower than rates previously reported using the same method.^11^

To investigate genetic landscape, we initially identified single nucleotide variations (SNVs) in various tissues and single cells. Deep sequencing of white blood cells served as the control. To minimize errors associated with principal component analysis (PCA) during the multiple displacement amplification (MDA) process, SNVs detected in any tissue or in at least three single cells were further analyzed as previously described.^12^ Altogether, 117 single nucleotide somatic mutations were identified (Supplementary Table 3–5), with an average of 39.82 mutations per single cell from either primary or metastatic tumor tissue, 10.01 mutations per cell from adenoma, and 0.65 mutations per normal cell (from blood and normal colon). The average somatic mutation rate in cancer cells was 0.85 per million bases (Mb), significantly higher than in the adenoma, which exhibited a rate of 0.02 per Mb.

### Clustering and PCA analysis on single cells

We performed clustering and PCA analysis on the single-cell exome sequencing data derived from the tumor, the adenoma, and normal cells. In the analysis, some cells from tumor tissues clustered tightly with the normal cells. These cells exhibited significantly fewer mutations, with only 1-2 mutations per cell, compared to other tumor cells. Moreover, they lacked harbor driver mutations typically associated with oncogenesis, such as mutations in the *APC* and *TP53* genes. It is likely that these cells represent normal stromal cells within the microenvironment of the tumor, including immune cells. Consequently, they were excluded from subsequent analyses of the cell populations. After removing the presumed normal cells, the malignant tissues exhibited an average of 43.23 mutations, which was considerably higher than the mutation rate observed in the adenoma (11.16 mutations per cell; *P* = 2e-16, unpaired *t* test, two-sided; Supplementary Fig. 3). The mutation counts between the two metastases and the different regions of the primary tumor showed no significant differences (*P* > 0.05, unpaired *t*-test, two-sided; Supplementary Fig. 3).

**Fig. 1:**
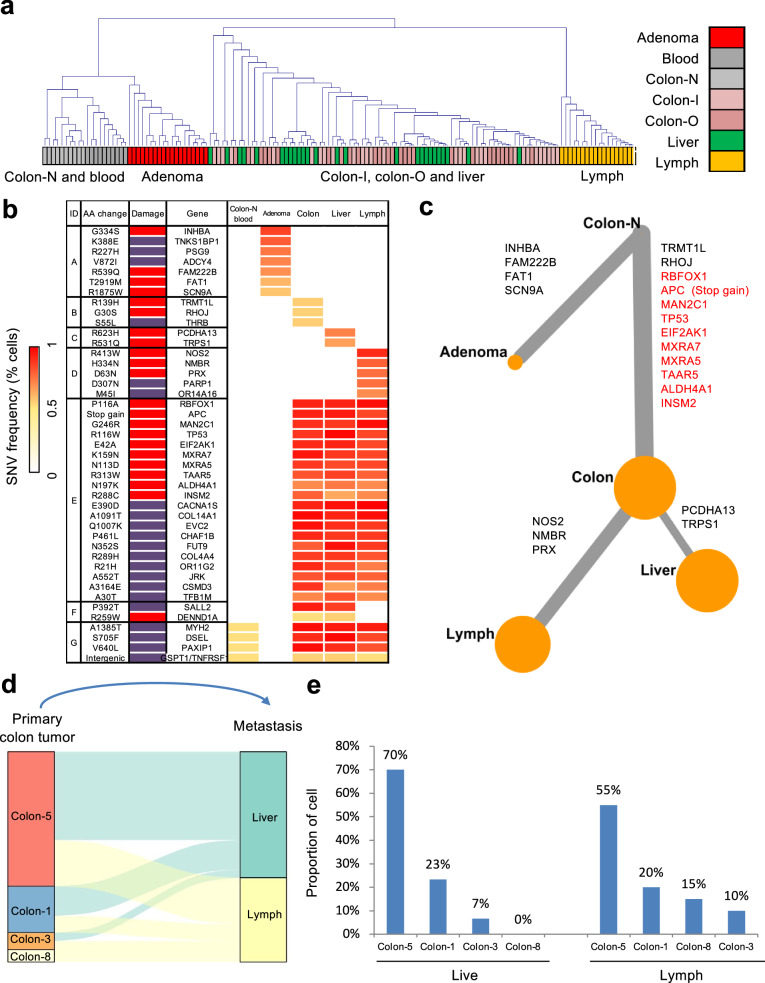
Clustering and evolution analysis of adenoma, primary colon cancer, and metastatic tissues from a stage IV colon cancer patient. Single cells in the liver and lymph metastases could be tracked from colon 5 or colon 1. **a** Clustering of exome sequencing data of 140 single cells from primary tumor, liver metastasis, lymphatic metastasis, and adenoma from a stage IV colon cancer patient. Colon-N, normal tissue from colon; Colon-I, inner circle colon cancer cells; Colon-O, outer circle colon cancer cells. The height of the vertical lines in the dendrogram of graphs reflects the level at which two clusters are merged (based on the similarity of single cell). The greater the height, the more dissimilar the two clusters were at the point of their merger. **b** Heat-map of the 7 groups of genetic alterations with different distributions among the tissues. A red block in the “Damage” column indicates an SNV predicted to damage protein function. A purple block indicates a tolerated SNV. **c** Phylogenetic tree reconstruction depicting the evolutionary trajectory of colon cancer. The size of the nodes reflects the SNV frequency at each evolutionary stage, branches width reflects the frequency of SNV gain or loss, and branches reflects the number of SNV changes at each stage. Gene mutations marked in red are also found in the liver and lymph metastases. **d** A Sankey diagram shows the possible origins of liver and lymph metastasis based on single-cell exome sequencing of different tissue sections from the primary colon tumor (colon-1, 3, 5, 8). **e** The proportion of cells supporting the possible sites of origin of liver and lymph metastasis

Based on these mutations, we were able to distinctly categorize the tumor and normal tissues into four groups: non-neoplastic, adenoma, primary tumor and liver metastasis, and lymphatic metastasis. Single cells from the normal blood and colon clustered together (Fig. 1a). Similarly, cells from the primary tumor and the liver metastasis grouped together, yet displayed distinct clustering patterns compared to cells from the lymphatic metastasis (Fig. 1a). The adenoma tissue exhibited a unique set of mutations and a distinct mutational signature distinct compared to the other tissues. In contrast, the primary tumor shared 50 mutations with both the liver and lymphatic metastases (Supplementary Fig. 4). Between the primary tumor and the liver metastasis, there were 67 common mutations, with only 6 and 3 unique mutations found in each tissue, respectively. The lymph node tissue displayed 16 unique mutations not observed in the primary tumor or the liver metastasis (Supplementary Fig. 4).

To determine the genetic characteristics that could differentiate the cells into distinct clusters in the PCA analysis, we clustered the single cells with mutations using an unsupervised hierarchical method (Fig. 1b), in which the tissues were separated in a similar manner as in the PCA analysis. Based on this classification, we pinpointed the most specific mutations in each tissue type (Fig. 1b) and elucidated the clonal evolution of this colon cancer case (Fig. 1c). The majority of the mutations, including the well-known driver mutations, had accumulated prior to metastasis (Fig. 1c and Supplementary Fig. 4). Notably, a few additional mutations were exclusively identified in cells from the liver or lymphatic metastasis, but not in the primary tumor tissues (Fig. 1c).

### Intra-tissue heterogeneity of the primary and metastatic tissues

Single cells were isolated from four distinct areas within the primary tumor (Supplementary Fig. 4a). We analyzed the mutational status of each single cell from the liver and lymphatic metastases, comparing these with all other single cells obtained from the primary tumor to identify cells exhibiting high similarity. Remarkably, 93% of the single cells in the liver metastasis were traceable to either colon 1 or colon 5, both located in the first quadrant of the primary tumor (Supplementary Fig. 4a). In contrast, only 7% of the single cells from the liver metastasis showed similarity to cells from the other regions in the primary tumor (*P* = 4.3e−05, Fisher’s exact test, two-sided) (Fig. 1d, e). Similarly, the majority of the single cells from the lymphatic metastasis showed similarity with cells from colon 1 and colon 5 (Fig. 1d, e). In this case, it appears that both the liver and lymphatic metastases could have originated from tumor cells in the region containing colon 1 and colon 5 (Supplementary Fig. 5). A study showed that in 65% of cases, liver and lymphatic metastases in colon cancer stem from distinct origins, whereas in 35% of cases, they arise from a common origin.^5^ The case in our study aligns with the latter scenario of a common origin. Furthermore, we found that the mutations distinguishing the lymphatic metastasis from the primary tumor were absent in the liver metastasis. This result indicated that the liver metastasis mainly arose directly from the primary tumor rather than through a lymphatic lineage (Fig. 1c).^5^

**Fig. 2:**
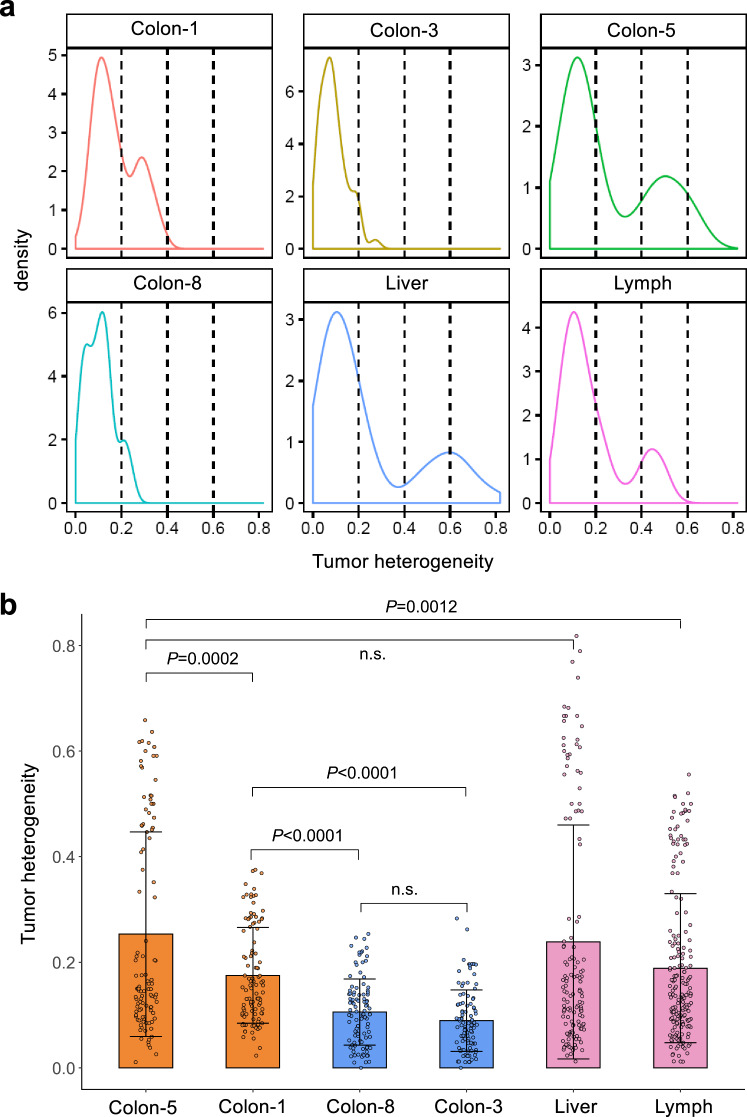
Intra-tissue heterogeneity of the primary and metastatic tissues based on single-cell mutation landscape. **a** A density curve showing the distribution of intra-tissue heterogeneity within the primary and metastatic tissues. **b** A bar plot showing the difference in heterogeneity between the primary and metastatic tissues. Unpaired *t*-test, two-sided

**Fig. 3:**
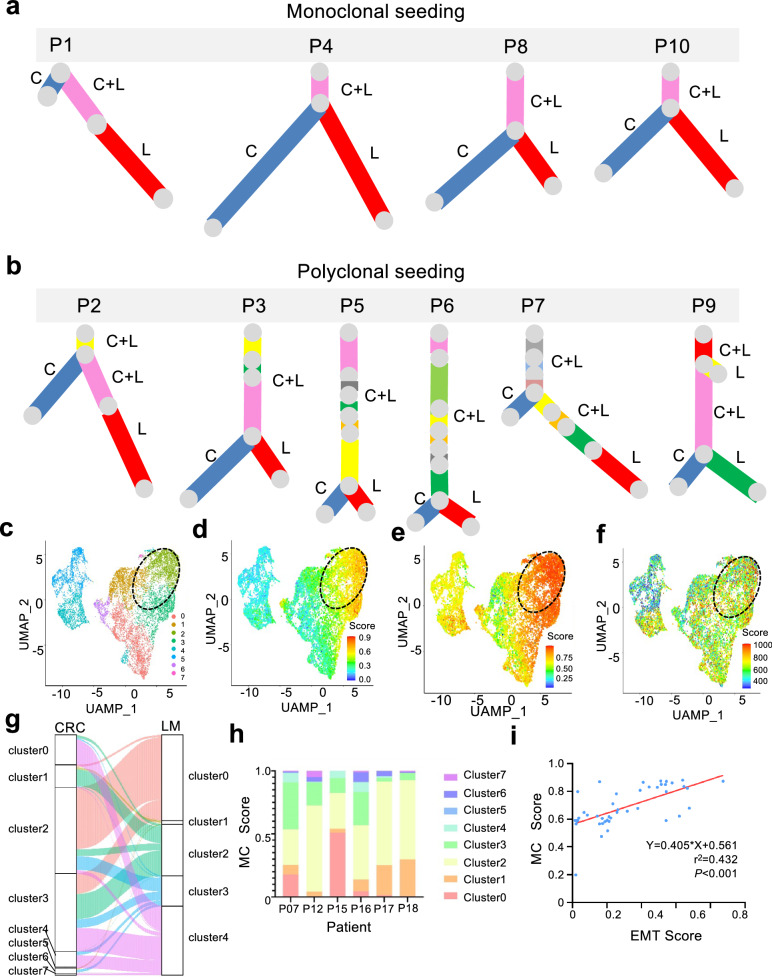
Subclonal structure within 10 metastatic colon cancers and single-cell RNA-seq analysis of primary CRC and matched liver metastases samples of 6 CRLM patients. **a** Monoclonal seeding of 4 patients is shown as phylogenetic trees. **b** Polyclonal seeding of 4 patients is shown as phylogenetic trees. C: colon cancer (primary tumor), L: liver metastases, C + L: colon cancer and liver metastases. The relative branch length of the phylogenetic tree reflects the percentage of all mutations in a cluster. **c** UMAP plot segregating cancer cells of primary CRC into 8 subgroups. Colors indicate different subgroups. **d**–**f** UMAP plot showing the MC score, EMT score, CNV score of each single cell. Cells are colored according to MC score. **g** Sankey plots showing the associations between subgroups of cancer cells in primary CRC and matched liver metastases. **h** Histogram showing the proportion of tumor cells in primary CRC that metastasized to the liver in each CRLM patient. **i** Scatter plot with trend line showing a significant positive linear correlation between the EMT score and MC score

We next analyzed the intra-tissue heterogeneity by calculating the similarity between every pair of single cells within the same tissue (see methods). Intriguingly, both the liver and lymphatic metastases showed higher levels of heterogeneity than the primary tumor. Among the four pieces of tissue from the primary tumor, colon 1 showed a partial similarity to the two metastatic tissues regarding the value and distribution of heterogeneity in the analysis, while colon 5 showed the greatest similarity to the liver and lymphatic metastases (Fig. 2a, b and Supplementary Fig. 6-8). These results are consistent with the prior analyses, showing that the metastatic tissues derived from tumor cells located in colon 1 and colon 5 of the primary tumor. The increased heterogeneity in metastatic tissues challenges the model where a single tumor cell migrates to the liver or lymph node, subsequently forming a metastatic lesion with less heterogeneity than the primary tumor. Instead, the results support the model in which a cluster of tumor cells originating from a primary tumor migrates to the liver and establishes liver metastasis. The group of migrating cells likely mirrors the heterogeneity observed in the primary tumor, which is then reflected in the liver metastasis. Consequently, the heterogeneity in the metastasis would resemble that of a specific region in the primary tumor, rather than being more heterogeneous due to aggregation of CTCs in the bloodstream, or being less heterogeneous due to seeding by a single cell. Further analysis of the exomes from the primary and liver metastatic tumors in 10 colon cancer cases revealed polyclonal seeding in 6 of these cases (Fig. 3a, b). We compared the clinical information of colorectal cancer patients in the monoclonal group (P1, P4, P8, P10) and the polyclonal group (P2, P3, P5, P6, P7, P9), including age, gender, and comorbidity, the response to preoperative chemotherapy, poor differentiation, T stage, lymphatic metastasis, microvascular invasion. Interesting, we found significant differences in the T stage between monoclonal and polyclonal groups (*P* < 0.05), with a higher proportion of polyclonal colorectal cancer patients in stage T3, and a higher proportion of monoclonal colorectal cancer patients in stages 4a and 4b. No significant differences were observed in the other clinical characteristics (*P* > 0.05, Supplementary Table 4). Our results suggest that there is no significant correlation with treatment, and further validation with a larger sample is required.

To further verify polyclonal seeding, we carried out single-cell RNA-seq analysis on primary CRC and matched liver metastases samples from 6 CRLM patients. We obtained 8 and 5 cancer subgroups in primary CRC and corresponding liver metastases samples, respectively (Fig. 3c, Supplementary Fig. 9a, b). We found the metastatic contribution score (MC score) of clusters 2 and 3 was significantly higher than those of the other clusters (Fig. 3d). Therefore, we calculated the EMT score of different subgroups, finding that clusters with high MC scores also exhibited elevated EMT scores (Fig. 3e). In addition, clusters with high MC scores also showed a high genomic instability (high CNV score) (Fig. 3f, Supplementary Fig. 9c). Utilizing the Sankey diagrams, we further demonstrated the high MC scores of subgroups 2 and 3 in primary cancer cells (Fig. 3g, h, Supplementary Fig. 9d-i), which exhibited a direct correlation with the MC score (*P* < 0.001, R^2^ = 0.432, Fig. 3i). This indicated that polyclonal tumor cells with high EMT-related gene expression were more likely to spread from the primary site to the liver. GO and KEGG analyses suggested that subgroups with high MC scores were more associated with EMT-related pathways (Supplementary Fig. 10). Additionally, to further explore the relationship between EMT scores and clinical characteristics at the single-cell level, we have newly collected six fresh tumor samples from liver metastases for single-cell transcriptome sequencing. These samples were obtained from six patients diagnosed with colorectal cancer liver metastases at the Cancer Hospital of the Chinese Academy of Medical Sciences. We discovered that patients accompanied with lymph node metastasis, patients with comorbidities, and patients with microvascular invasion (MVI) had higher EMT scores (Supplementary Fig. 11; all p ≤ 2e−16). Patients in stage T4 had higher EMT scores compared to those in stage T3 (p ≤ 2e-16). Patients who showed a positive response to preoperative chemotherapy had lower EMT scores compared to those who did not respond (p ≤ 2e−16).

**Fig. 4:**
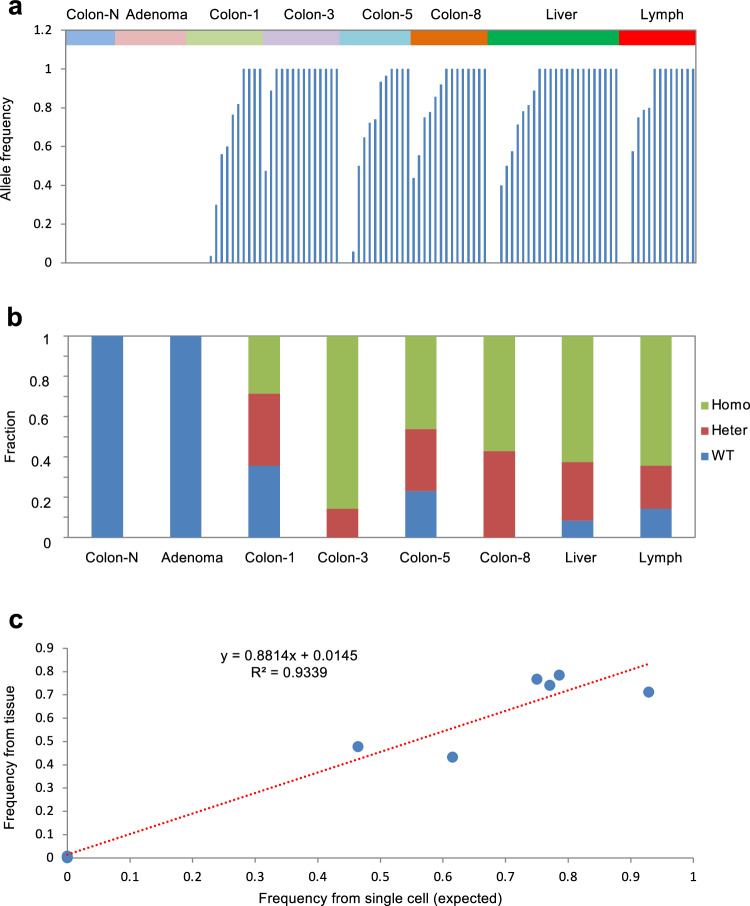
Intra-tissue heterogeneity of the primary and metastatic tissues based on the mutation status of *APC*. **a** The allele frequency of the *APC* stopgain mutation in the single cells from normal tissue, adenoma, colon-1, colon-3, colon-5, colon-8, liver metastasis and lymphatic metastasis. **b** The distribution of single cells categorized by homozygous mutant, heterozygous mutant, and wild-type *APC*. **c** The fractions of the *APC* mutation calculated from single cells correlate directly with mutation status based on the bulk tissue sequencing

To validate the heterogeneity estimated by the statistics of all mutations, we focused the analysis on the mutation status of the *APC* gene. In each single cell, the mutation status can be classified as homozygous wild-type, homozygous mutant or heterozygous. The homozygous *APC* mutants could exhibit a stop-gain mutation in one allele and loss of heterozygosity (LOH) in the other. A heterozygous mutant could display a stop-gain mutation in one allele alongside a wild-type *APC* allele, which could be inactivated by alternative mechanisms such as DNA methylation within the promoter sequence. The single cells in colon 3 and colon 8 were dominated by both homozygous and heterozygous mutant cells. In contrast, colon 1, colon 5, and the two metastatic tissues displayed greater heterogeneity in the *APC* mutation status (Fig. 4 and Supplementary Figs. 12–14). A significant percentage of single cells were of homozygous wild-type at the position of the *APC* stop-gain mutation. These cells, while appearing normal, were integrated within the tumor tissue and harbored additional mutations. The absence of the stop-gain mutation could not be explained by allele dropout during the whole genome amplification (WGA) process based on the evaluation of the occurrence frequency of allele dropout in this study. Furthermore, we evaluated the mutation fraction of the *APC* mutation by combining the mutation status across single cells within a given tissue. We found that the mutation fractions derived from single-cell analyses correlated directly with those obtained from bulk tissue sequencing. Notably, these *APC* wild-type single cells showed a different mutation status than the *APC* mutant cells. Moreover, such *APC* wild-type single cells showed a similar distribution in colon 1, colon 5, and the two metastatic tissues. The heterogeneity analysis based on the status of a single *APC* stop-gain mutation also revealed a similar result to that based on the genetic landscape of a single cell, indicating that the metastatic tissues were derived from colon 1 and colon 5. This suggests that the tissue migration likely involved not just a single cell but a cluster of cells, including both *APC* wild-type and mutant cells.

### TRPS1 R544Q mutation facilitates colorectal cancer metastasis in vitro and in vivo

Mutational discrepancies between primary tumors and their metastases can highlight crucial driver genes involved in metastatic progression, offering a deeper understanding of the underlying biology of metastasis. The TRPS1 R544Q mutation was compellingly indicated in the single cells from the liver metastasis, but not in cells from the primary tumor or the lymphatic metastasis. Given that *TRPS1* mutations have been previously reported in human cancers and associated with metastasis, *TRPS1* was singled out as a candidate gene for additional investigation into the molecular mechanisms responsible for distant metastasis in CRC.

**Fig. 5:**
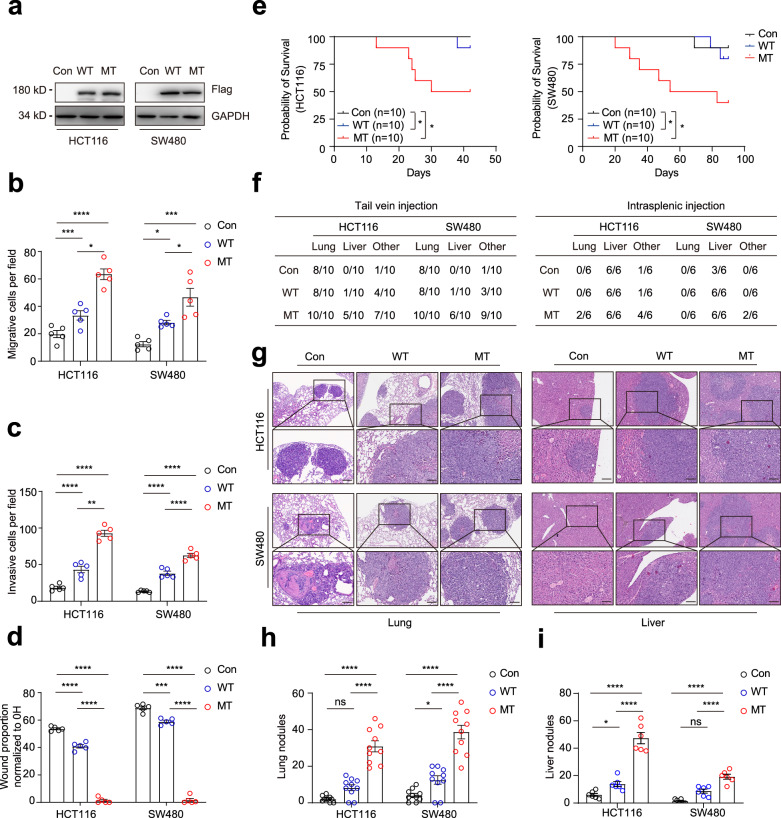
*TRPS1* R544Q mutation promotes colorectal cancer cells metastases in vitro and in vivo. **a** Western blot analyses of ectopic expression of *TRPS1* and its mutant in HCT116 and SW480 cells. GAPDH serves as a loading control. (**b**, **c**) Graphs showing numbers of migrating or invasive cells expressing *TRPS1* in HCT116 and SW480 cells using **b** transwell migration and **c** Matrigel invasion assays. **d** Bar graphs showing proportion of wound healing assay after 36 hours in the indicated HCT116 and SW480 cells. **e** Kaplan-Meier survival curves for BALB/c nude mice and NSG mice injected via tail vein with indicated HCT116 (n = 10) and SW480 cells (n = 10). **f** Table showing the incidence of metastasis in the lung, liver and other organs in mice. Other, additional organ metastases. **g** Representative images stained with H&E of lung (left) from tail vein models and liver (right) section from splenic intravenous injection models, featuring HCT116 (top) and SW480 (bottom) cells. Scale bars, 100 µm. Quantification of the number of **h** lung and **i** liver colonizing foci from the different groups. Data are represented as the mean ± SEM. Empty vector: Con, *TRPS1* wild type: WT, *TRPS1* mutant (R544Q): MT. All data are represented as the mean ± SEM, ns, not significant, *P < 0.05, **P < 0.01, ***P < 0.001, ****P < 0.0001

Firstly, *TRPS1* expression was examined in seven CRC cell lines, revealing low expression levels in HCT116 and SW480 cells (Supplementary Fig. 15a). To further investigate, we created HCT116 and SW480 cell lines with stable ectopic expression of both wild-type and mutant *TRPS1* (empty vector, Con; wild type, WT; and R544Q mutant, MT), which was confirmed by Western blot and Sanger sequencing (Fig. 5a, Supplementary Fig. 15b). We next examined the distant organ homing potential of mutant *TRPS1* in migration and invasion assays in vitro. The results demonstrated that the metastatic ability of the TRPS1-MT group was 2 to 3 times higher than that of the TRPS1-WT group, and approximately 3 to 5 times higher than that of the control group (Fig. 5b, c, Supplementary Fig. 15c). In wound healing assays, linear scratch wounds inflicted on TRPS1-MT cells completely healed within 36 hours post-injury, whereas the wound area in the control and TRPS1-WT cells exhibited only about 30 to 60% healing during the same period (Fig. 5d, Supplementary Fig. 15d).

To examine metastatic potential of TRPS1-MT in HCT116 and SW480 cells, we established two mouse models of lung and liver metastases by tail vain vein and intrasplenic injections, respectively. Mice injected with TRPS1-MT cells showed substantially shorter survival rates than those with TRPS1-WT or control cells (Fig. 5e). Furthermore, the mice bearing TRPS1-MT cells developed extensive metastases across multiple organs, including the lung, liver, heart, pancreas, ovary and distant lymph nodes (Fig. 5f, Supplementary Fig. 15e). However, metastases occurred with less frequency in mice injected with TRPS1-WT and control cells, with the lowest frequency observed in the control group (Fig. 5f). Hematoxylin and eosin (H&E) staining of sections from lung (from tail vein injections) and liver (from intrasplenic injections) tissues showed that the number of nodules in the TRPS1-MT group was about three to five times greater than that in the TRPS1-WT group, while the control group had relatively fewer nodules (Fig. 5g–i). Ki67 staining showed that *TRPS1* mutation showed obviously cell proliferation in liver metastases (Supplementary Fig. 15f). Similar metastatic results were observed in Luciferase-labeled HCT116 cells following *TRPS1* mutant overexpression (Supplementary Fig. 15g). Taken together, TRPS1 R544Q mutation enhanced metastasis of HCT116 and SW480 cells both in vitro and in vivo.

### *TRPS1* mutant (R544Q) promotes epithelial to mesenchymal transition through binding to the promoter region of *ZEB1*

**Fig. 6:**
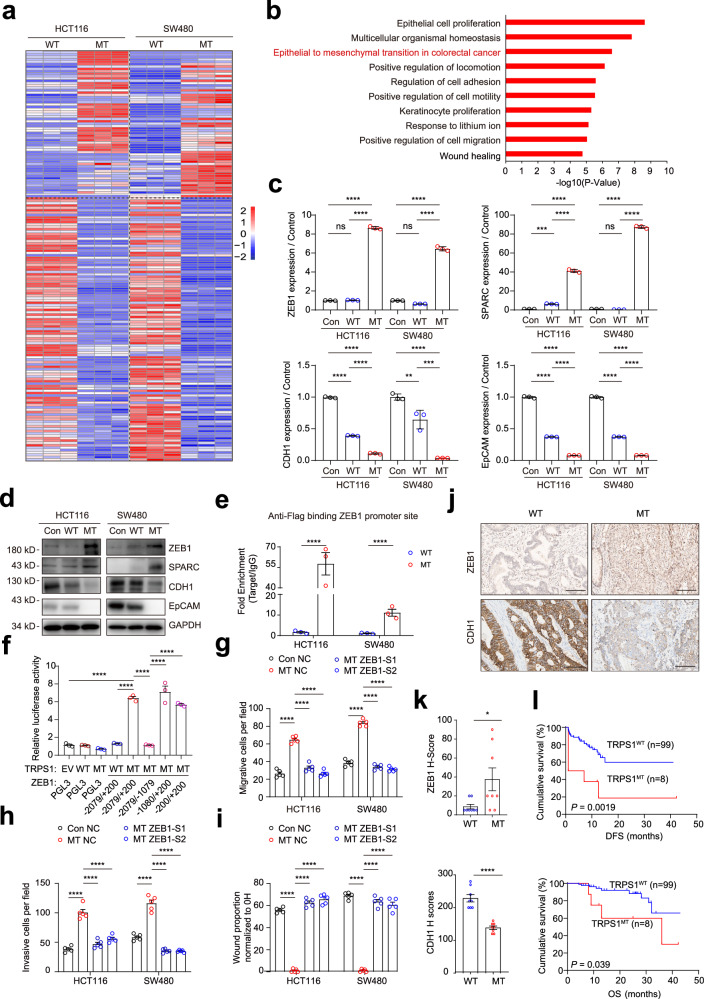
TRPS1 mutant (R544Q) promotes the colorectal cancer cell metastases through ZEB1. **a** Heatmap showing 172 common DEGs between HCT116 WT versus HCT116 MT (left) and SW480 WT versus SW480 MT (right) cells. Red and blue indicate high and low expression of genes. **b** The histogram of significantly enriched biological processes represented by the 172 DEGs from **a** generated through metascape functional enrichment analysis. **c** qRT-PCR and **d** western blot analysis showing mRNA and protein levels of ZEB1, SPARC, CDH1, and EpCAM in the indicated HCT116 and SW480 cells. **e** ChIP-qPCR analysis of *TRPS1* enrichment at the *ZEB1* promoter region in cells expressing *TRPS1* WT and MT (R544Q). **f** Dual-luciferase reporter assay for co-transfection of *TRPS1* WT/MT expression plasmids with *ZEB1* promoter luciferase reporter constructs in 293 T cells. *ZEB1* promoter fragments include the region from -2079/+200 and three truncated fragments (truncated fragment 1 [-2079~−1079], truncated fragment 2 [−1080~+200], truncated fragment 3 [−200~+200]. Bar graphs showing the number of migrating and invasive cells for HCT116 and SW480 cells with *TRPS1* mutant/control and *ZEB1* knockdown in **g** transwell migration and **h** Matrigel invasion assays. **i** Bar graph representing proportion of wound healing at 36 hours in HCT116 and SW480 cells with *TRPS1* mutant/control and *ZEB1* knockdown. **j** Representative images of immunohistochemistry for ZEB1 (upwards) and CDH1 (below) in CRC samples with *TRPS1* wild type and mutations. Scale bars, 100 µm. **k** Quantification of ZEB1 (upwards) and CDH1 (below) H-scores in CRC samples with *TRPS1* WT and all *TRPS1* mutations. **l** Kaplan–Meier survival curves showing the correlation between *TRPS1* mutant and DFS (upwards) or OS (below) in 107 CRC samples. Data are represented as the mean ± SEM. Empty vector: Con; *TRPS1* wild type: WT; *TRPS1* mutant (R544Q): MT. All data are represented as the mean ± SEM, ns, not significant, *P <0.05, ****P < 0.0001

To investigate the molecular mechanisms underlying CRC metastasis mediated by the *TRPS1* R544Q mutation, we next performed bulk RNA sequencing on modified HCT116 and SW480 cells. The analysis revealed a total of 172 differentially expressed genes (DEGs) in common between HCT116 and SW480 TRPS1-WT and TRPS1-MT cells (Fig. 6a, Supplementary Table 6). Subsequently, we performed metascape analysis highlighted significant enrichment in pathways including epithelial cell proliferation, epithelial to mesenchymal transition (EMT) in colorectal cancer, regulation of cell adhesion, positive regulation of cell motility and positive regulation of cell migration (Fig. 6b). Given the critical role of EMT in cell proliferation, propagation, plasticity, invasion and migration,^13–15^ we focused on the function of 20 EMT-associated genes. Among these, *ZEB1*, *SPARC*, *CDH1* and *EpCAM* were the most significantly differentially expressed genes between TRPS1-WT and TRPS1-MT cells and had been previously associated with colorectal cancer metastasis^16–19^ (Supplementary Fig. 16a). The qRT-PCR analysis showed upregulation of *ZEB1* and *SPARC* and downregulation of *CDH1* and *EpCAM* compared with TRPS1-WT or control cells (Fig. 6c, Supplementary Table 7). Western blot analysis confirmed these results (Fig. 6d). Conversely, knocking down *TRPS1* resulted in decreased expression of *ZEB1* and *SPARC*, while *CDH1* and *EpCAM* levels were recovered (Supplementary Fig. 16b). As ZEB1 was considered as an important transcription factor to increase the epithelial-mesenchymal transition and associated with the regulation of CDH1 and EpCAM genes. Then we silenced *ZEB1* expression, the other three genes (CDH1, SPARC, and EpCAM) were reverted (Supplementary Fig. 16c).

As TRPS1 is a transcription factor mainly located in the cellular nucleus (Supplementary Fig. 16d), we performed a Cleavage Under Targets and Tagmentation (CUT&Tag) sequencing assay on TRPS1-WT and TRPS1-MT cells to delineate the whole-genome binding sites of *TRPS1*. Our results revealed an enrichment of various EMT motifs associated with *TRPS1* mutation. Through the analysis including RNA sequencing, we identified eight overlapping genes, including *ZEB1* and *EpCAM*, regulated by mutant *TRPS1* in both HCT116 and SW480 cells (Supplementary Fig. 16e). Further validation was achieved through ChIP-qPCR, confirming the binding of TRPS1-MT to the promoters of *ZEB1* and EpCAM. Notably, TRPS1-MT exhibited specific binding to the *ZEB1* promoter, unlike TRPS1-WT, which showed no effective binding. In contrast, there was no significant difference in binding to the *EpCAM* promoter between TRPS1-WT and MT cells (Fig. 6e, Supplementary Fig. 16f, g). Therefore, ZEB1 was regarded as a key target for TRPS1-MT in CRC hepatic metastases. In the next luciferase reporter assays, TRPS1-MT significantly enhanced luciferase activity of the *ZEB1* promoter constructs, specifically within the -200 ~ +200 region (Fig. 6f). These data indicated that *ZEB1* was a candidate target of the *TRPS1* R544Q mutant in driving colorectal cancer metastasis. To further demonstrate *ZEB1* as a downstream target of *TRPS1* in tumor cell metastasis, we examined the effects of *ZEB1* knockdown on migration, invasion and wound healing of TRPS1-MT cells (Fig. 6g–i, Supplementary Fig. 16h, i). The results demonstrated that knockdown of *ZEB1* significantly inhibited cell migration and invasion induced by the *TRPS1* R544Q mutant.

Next, we performed immunohistochemical (IHC) staining on genome-sequenced primary human colorectal cancers tissues to evaluate the correlation between the expression of *ZEB1*, CDH1 and *TRPS1* mutation. The result demonstrated that *ZEB1* expression was pronounced in CRC tissues with *TRPS1* mutation (n = 8) than in *TRPS1* wild type samples (n = 8), while the CDH1 expression was diminished in the CRC tissues with *TRPS1* mutation (Fig. 6j, k). We then analyzed the correlation between *TRPS1* mutation status and pathological stages of 107 CRC tumors (Table 1, Supplementary Table 8). The results showed a significant association between *TRPS1* mutations and stage M1 (Fisher’s exact test, *P* = 0.0314). More importantly, the Kaplan–Meier survival analysis revealed that *TRPS1* mutation closely correlates with poor disease-free survival (DFS) (*P* = 0.0019) and worse overall survival (OS) (*P* = 0.039), highlighting its prognostic relevance in CRC patients (Fig. 6l).

**Table 1:** Relationship between *TRPS1* status and pathological stages

Pathological parameters	N	*TRPS1* status		*P* value
		wide type	mutation	
T stage				0.6679
T1	0	0	0	
T2	9	9	0	
T3	63	58	5	
T4	35	32	3	
N stage				0.7498
N0	37	35	2	
N1	41	38	3	
N2	29	26	3	
M stage				0.0314*
M0	54	53	1	
M1	53	46	7	
AJCC stage				0.1636
I	6	6	0	
II	13	12	1	
III	32	32	0	
IV	56	49	7	

^*^P value < 0.05

To further validate of the association between the gene expression of *TRPS1* and EMT in CRC patients, we analyzed 594 colorectal samples from the TCGA dataset, and found that *TRPS1* expression showed a weak correlation with KRAS, NRAS, HRAS and BRAF (Spearman=0.24, 0.13, -0.24, 0.25, all p-values < 1e−9, Supplementary Fig. 17a–d), but discovered a significant direct relationship with *ZEB1* (Spearman=0.76, p-values = 3.27e−114, Supplementary Fig. 17e) and with *ZEB2* (Spearman=0.79, p-values = 3.32e−129, Supplementary Fig. 17f).

Collectively, these results demonstrate that the *TRPS1* mutation significantly promotes colorectal cancer metastasis by activating *ZEB1* expression, thereby enhancing epithelial to mesenchymal transition. This mechanistic pathway ultimately leads to poor prognosis in patients with CRC.

## Discussion

CTC clusters were first observed in 1960 and have repeatedly been connected to poor prognosis and cancer progression.^11,20,21^ Previous studies have shown that CTC clusters are predominantly formed as a cohesive group of tumor cells originating directly from the primary site, rather than forming through intravascular aggregation.^22^ Such CTC clusters have demonstrated the ability to reorganize their geometries and successfully navigate through 5-μm constrictions, indicating their potential to traverse capillaries and reach metastatic organs.^23^ However, these results were primarily based on animal studies, and there remains ongoing debate regarding whether these clusters originate from a monoclonal or polyclonal source within the primary tumor, and their exact role in promoting metastasis.^24–27^ Multiple competing models of metastasis have been reported, including monoclonal seeding and polyclonal seeding.^28^ Our findings, which compare heterogeneity and mutation status between single cells from primary tumors and their corresponding metastases, lend support to the model suggesting that both liver and lymph metastases are derived from CTC clusters directly released from the primary tumor, rather than from solitary CTCs or clusters formed via intravascular aggregation and proliferation of individual CTCs. Furthermore, our results indicate that the CTC clusters, which contribute to liver and lymphatic metastases, comprise a relatively large number of tumor cells. Thus, the metastatic nodules are capable of preserving the heterogeneity observed in the primary tumor. In addition, our results show that the metastatic ability of primary tumor cannot be inferred based solely on the location within the colon (Colon-I, inner circle colon cancer cells vs. Colon-O, outer circle colon cancer cells). Further results indicate that the heterogeneity of primary tumor plays a critical role in its metastatic ability, a notion corroborated by findings in the *APC* gene model. Besides, single-cell transcriptome analysis demonstrated polyclonal tumor cells with high EMT gene expression were more likely to disseminate from the primary site and establish in the liver (Supplementary Table 9).

Distant metastasis, particularly to the liver, is the principal factor contributing to death in cancer patients. Certain driver gene mutations may not only confer metastatic potential, but also provide viable therapeutic targets. Thus, there is an urgent need to identify the drivers of CRC metastasis, focusing on the mechanisms of hepatic homing and colonization. Here, we performed single-cell exome sequencing to investigate the *TRPS1* R544Q mutation in monophyletic seeding cases to determine its prevalence in CRC liver metastasis.^29^ *TRPS1* is known for its role in tricho-rhino-phalangeal syndrome, a hereditary autosomal dominant condition affecting hair follicles as well as craniofacial and skeletal development, and its dysregulation can lead to severe developmental anomalies.^30^ While *TRPS1* overexpression is commonly observed in breast carcinomas, ovarian cancer, osteosarcomas and endometrial cancer, indicating oncogenic properties, there are also several studies that suggest its tumor suppressor activities.^31–34^ Our study showed the *TRPS1* R544Q mutation, a novel point mutation, dramatically promoted the motility and invasive properties of colorectal cancer cells. Together, these biological properties underlie the significantly greater colonization of the liver and extrahepatic organs observed in mice injected with cells containing the *TRPS1* R544Q mutation, which also exhibited shorter survival time compared with those injected with cells expressing *TRPS1* wild-type or the control. In addition, *TRPS1* mutations were identified in 8 out of 107 CRC patients characterized by advanced metastatic stages and poor prognosis. Finally, TRPS1 was shown to moderately enhance migratory potential both in vitro and in vivo, underscoring its oncogenic role. Collectively, these results suggest that *TRPS1* mutations, especially the *TRPS1* R544Q mutation, function as oncogenic mutations and may act as prognostic indicators in individuals with CRC metastases.

Dysregulation of EMT signaling is frequently observed in the progression of cancer aggressiveness.^35^ ZEB1, a key transcription factor of EMT, is often upregulated during carcinogenesis, thereby activating EMT pathways.^36^ TRPS1, an atypical GATA transcription factor, has been identified as a key regulator of the mesenchymal-to-epithelial transition, affecting the formation and specialization of various tissues.^37–39^ Despite its known roles, the mechanism by which the *TRPS1* R544Q mutation influences EMT signaling to enhance the metastatic phenotype in colorectal cancer remains unknown. In our research, we identified *TRPS1* R544Q acts as an activator of EMT signaling via upregulating both mRNA and protein levels of *ZEB1* in cells harboring the mutated *TRPS1*. In contrast, silencing of *ZEB1* in CRC cells with *TRPS1* mutations diminished the aggressive phenotype, supporting the notion that *TRPS1* R544Q mutation promoted colorectal cancer metastasis through transcriptional activation of ZEB1. Our study demonstrated that *TRPS1* R544Q likely binds to the *ZEB1* promoter sequence (-200 ~ +200 region), although additional research is necessary to fully clarify the underlying mechanisms. Finally, our patient data also confirmed that *ZEB1* overexpression is associated with the *TRPS1* mutation. Collectively, these results illuminated the *TRPS1* R544Q mutation as a crucial transcription factor that activates *ZEB1* expression, thereby driving CRC metastasis.

Due to the fact that we only analyzed a sample from one patient, there are inherent limitations due to individual-specific factors, which restrict the generalizability of our findings. We are unable to perfectly classify a single sample directly, but we have further investigated the relationship between colorectal cancer patients with *TRPS1* gene mutations and several key molecular subtypes of colon cancer, validating our findings through comprehensive multi-omics analysis. Firstly, at the single-cell genomic scale, we conducted exome sequencing on 150 cells from 9 tissue samples. Secondly, on the bulk genome level, we performed whole-exome sequencing on the primary colon tumor and matched liver metastasis tissues from 10 patients, substantiating the theory that the liver metastases arose from polyclonal seeding of the primary tumors. Thirdly, at the single-cell transcriptome level, we analyzed transcriptome sequencing data from primary colon cancers and matched metastasis tissues of 6 patients. Our findings indicated that tumor cells exhibiting elevated expression of EMT-related genes were more prone to disseminate from the primary site and establish in the liver, thereby strengthening our conclusions. This multi-faceted approach not only strengthens the credibility of our findings but also underscores the complex nature of cancer metastasis, offering deeper understanding of tumor dissemination and identifying potential avenues for treatment.

In summary, our study conducted a comprehensive genetic profiling of a substantial number of single cells obtained from the primary tumor and metastatic sites in a colon cancer patient. Single-cell level profiling allowed us to compare the genetic landscapes of different tissues and to examine the intra-tissue heterogeneity within each sample. Our results support the model that both liver and lymphatic metastases are derived from a specific region within the primary tumor and involve the migration of independent polyclonal cell clusters. Finally, TRPS1 may promote metastatic polyclonal seeding in colon cancer by enhancing epithelial-to-mesenchymal transition.

## Materials and Methods

### Human CRC clinical tissues

The paraffin-embedded CRC specimens and the corresponding evaluation of the histomorphology were obtained from the Department of Pathology. Clinical parameters were obtained from the Department of Hepatobiliary Surgery. The characteristics of the patients are detailed in Table 1.

### Single-cell exome sequencing and mutation analysis

Tissues (n = 9) from a 76-year-old male stage IV colon patient were sequenced with a mean fold depth of 108.6 and 99.2% coverage (Supplementary Table 1 and Supplementary Fig. 1). Single cells from the tissues were lysed, and whole-genome amplification of the DNA was performed with multiple displacement amplification (MDA). Genomic libraries were constructed from the amplified DNA and the coding regions were enriched using the Agilent SureSelect Human All Exon 50 M platform (Supplementary Table 2-3 and Supplementary Fig. 1). The exome libraries were sequenced on the Hiseq 2000 platform (Illumina, San Diego, CA, USA).

Classical SNV detection algorithms were not supported by the ADO parameter. In addition, there were some SNVs that did not achieve sufficient coverage depth for SNV calling. In this case, a Bayes method was used to take both ADO and sequence data into consideration to estimate the genotype at SNV sites, assuming a diploid genotype at each SNV site. After Bayes estimation, the mutant allele frequency across the single-cell population was compared with the frequency observed in the deep tissue sequencing. All SNVs were supported by at least 10 sequencing reads in at least one tissue and 3 single cells. This high correlation also indicated that SNV calling followed by Bayes estimation exhibited good performance with a low false discovery rate.

### Heterogeneity analysis and comparison

To compare the evolutionary relationship and the heterogeneity of the single cells within and between the tumor tissues, the exact mutation status of each single cell was determined. The status of a mutation in a single cell was classified as mutant, wild type, or cannot determine (CND) due to insufficient coverage.

The similarity between every pair of single cells was carefully compared based on the mutation status. The similarity of two single cells was defined as follows. (a) We considered two single cells as the same (R = 1) only when all the mutation statuses were the same or at least one of them could not be determined. (b) If the two cells had different mutation statuses among all sites, the R value was −1. c) If the mutation status was wild type in one single cell and mutant in the other, this pair was deemed as different (R = -1). The similarity index of a tissue was the average R value of all the cell-cell combinations.

### Mutational signature analysis

The mutational signatures of 150 single cells were analyzed using the Wellcome Trust Sanger Institute mutational signatures framework with the non-negative matrix factorization (NMF) algorithm.^40^ Mutational signature analysis was performed using a 4-step process: a, 96-class mutational matrix was constructed, encompassing the 6 mutation types (T > C, T > G, T > A, C > G, C > T and C > A), along with the 5’ context (A, G, C, T), and 3’ context (A, G, C, T), derived from the mutation data of all samples. b, the number of operative processes in 150 single cells was determined by analyzing signature stability and Frobenius reconstruction errors across K = 1 to K = 15 signatures. c, mutational signatures (Sig A and Sig B) across all samples were identified using the NMF algorithm, reflecting the number of active processes established in step b. d, cosine similarity was used for unsupervised hierarchical clustering of the two mutational signatures (Sig A and Sig B) identified in our series, comparing them to the 30 mutational signatures (Sig 1-30) previously characterized in a pan-cancer study.^3^

### Subclonal structure analysis within 10 metastatic colon cancers

Mutations in 20 tissues (colon primary tumor and liver metastases tumor) from 10 colon cancer patients with liver metastasis were analyzed through whole exome sequencing. The method of whole exome sequencing and data analysis were performed as previous described.^41^ The data quality control and mutation of each sample are shown in Supplementary Table 4-5. Subclonal structure and phylogenetic trees were elucidated with the “pigeon-hole” principle within each patient.^42^ The branch lengths of the phylogenetic trees were determined from the proportion of all mutations within a cluster.

### Single-cell RNA-seq analysis

We obtained the single-cell RNA sequencing (scRNA-seq) data of colorectal liver metastases (CRLM) samples from the GEO database (GSE178318).^43^ The dataset included the primary CRC and corresponding liver metastases samples of six CRLM patients. We used the Seurat R package (version 4.2.2)^44^ for standard downstream processing of the sc RNA-seq data of the CRLM patients. The analysis excluded the genes detected in fewer than three cells, and cells containing fewer than 200 detected genes. The mitochondrial content was limited to less than 20%. Then, the data was normalized using the LogNormalize method. Principal component analysis (PCA) was used for unsupervised clustering, and the “JackStraw” function was applied to identify and visualise the selection of principal components. We employed nonlinear dimensionality reduction UMAP for clustering and applied the “FindAllMarkers” function to identify marker genes based on inter-cluster differences. The cell clusters were subsequently annotated according to known cell-specific marker genes. The “limma” R package was used to identify DEGs between clusters, with thresholds set at |log2 fold change (FC)| >1 and P < 0.01. The “clusterProfiler” R package was employed to conduct GO and KEGG enrichment analyses of the DEGs, with a threshold of P < 0.05 indicating significant enrichment. Epithelial cells were identified epithelial cells (EPCs) leveraging the higher expression of EPCAM and utilizing the InferCNV package to detect CNVs in EPCs and to recognize real cancer cells. The CNV score for each cell was determined by calculating the quadratic sum of CNV regions.^45^ The similarity between the primary lesion of CRC and the corresponding liver metastases was calculated using SCMAP method,^46^ and the extent of similarity was determined by the “metastatic contribution score (MC score)”. Then, we used the R package “ggalluvial” to construct a Sankey diagram. Based on previous studies, we defined the EMT characteristics and assessed the “EMT score” of each cell using the “AddModuleScore” function. The relevance between “contribution degree” and “EMT score” was examined using linear regression analysis. Histograms were employed to illustrate the distribution of tumor cell subsets migrating from the primary site to the liver in different patients.

To explore the correlation between EMT scores and clinical characteristics at the single-cell level, we collected six fresh tumor samples from liver metastases for single-cell transcriptome sequencing. These samples were obtained from six patients diagnosed with colorectal cancer liver metastases at the Cancer Hospital of the Chinese Academy of Medical Sciences. The analysis workflow for these samples is the same as the one described above for single-cell data analysis.

### Cell culture

The cell lines HCT116, SW480, 293T, HT29, SW620, and RKO cell lines were acquired from the Cell Resource Center, Peking Union Medical College (Beijing, China). HCT116, SW480, RKO, HT29, and SW620 were cultured in IMDM (Hyclone, SH30228.01, Logan, UT, USA), which was supplemented with 10% FBS (Gibco/Thermo Fisher Scientific, 10099141C, Waltham, MA, USA) and 1% penicillin-streptomycin (PS) (Gibco, SH30256.01). The cultures were maintained at 37 °C in an atmosphere containing 5% CO2. 293T cells were maintained in DMEM (Hyclone, SH30023.01), which was supplemented with 10% FBS and 1% PS, under the same conditions of temperature and CO2 concentration. Authentication of all cell lines was performed using STR profiling, and they were also tested for mycoplasma contamination.

### Plasmids and stable cell line constructions

The full-length human *TRPS1* gene (Accession: NM_014112.5) was cloned into a 3 × Flag expression pHBLV vector (GeneChem, Shanghai, China) to generate a TRPS1 overexpression plasmid. A *TRPS1* mutant (R544Q) was also constructed in the same vector with the Mut Express MultiS Fast Mutagenesis Kit (Vazyme, C215, Nanjing, China). All plasmids underwent verification through Sanger sequencing (TSINGKE, Beijing, China). Subsequently, the overexpression plasmid (including the empty vector [Con], TRPS1 wild-type vector [WT], and TRPS1 mutant vector [MT]) was co-transfected with packaging vectors psPAX2 and pMD2.G into 293 T cells for lentivirus production. Virus harvests were conducted at 48 h and 72 h post-transfection. Cell lines were infected with the lentiviruses and selected using puromycin (1 µg/mL, Selleck Chemicals, S7417, Houston, TX, USA) for two weeks. The selected cells were examined on a western blot and maintained to perform experiments.

### Western blotting

Cells were lysed using an SDS lysis buffer consisting of 50 mM Tris–HCl (pH 6.8), 10% glycerol, and 2% SDS, which was supplemented with a protease inhibitor cocktail (Roche, 11697498001, Basel, Switzerland). Protein quantification was performed using the BCA protein assay kit (Applygen, P1511, Beijing, China). Subsequently, the cell lysates were loaded onto an 8%-12% SDS–PAGE gel, fractionated, and then transferred to a PVDF membrane (Merck Millipore, IPVH00010, Burlington, MA, USA). The membranes were blocked with 5% (w/v) skim milk at room temperature for 1 hour and then incubated with primary antibodies overnight at 4 °C. Following this, the membranes were washed with TBST (3 × 10 min) and incubated with HRP-conjugated secondary antibodies (Zhongshan Golden Bridge Biotechnology, ZB2305, ZB2301, Beijing, China) for 1 hour at room temperature. Protein bands were visualized using an ECL blot detection system (Applygen, P1020). The primary antibodies employed included rabbit anti-TRPS1 (1:1000, NOVUSBIOLOGICALS, NBP2-04082, CO, USA), rabbit anti-EpCAM (1:1000, Proteintech, 21050-1-AP, Rosemont, IL, USA), rabbit anti-SPARC (1:1000, Proteintech, 15274-1-AP), mouse anti-CDH1 (1:1000, Cell Signaling, 14472, Danvers, MA, USA), rabbit anti-Flag (1:1000, Cell Signaling, 14793), rabbit anti-ZEB1 (1:1000, Cell Signaling, 3396), and mouse anti-GAPDH (1:3000, Proteintech, 60004-1-Ig).

### In vitro migration and invasion assays

The wound healing assay was conducted by plating cells (1 × 10^6^ HCT116 or SW480 per well) in a 6-well plate, with wounds created in a confluent monolayer using a pipette tip. Assessment was performed at 0 h and 36 h, with representative images captured at each time point. For the migration assay, 1 × 10^5^ HCT116 or SW480 cells in serum-free IMDM were seeded into the upper chamber of uncoated transwell inserts (Corning, 3422, Corning, NY, USA) featuring 8 μm pores. The lower chamber was filled with 600 μL of complete IMDM containing 10% FBS as a chemo-attractant. After 24 hours, cells were fixed with methanol and stained with 0.1% crystal violet at room temperature. Similarly, for the invasion assay, 1 × 10^5^ HCT116 or SW480 cells in serum-free media were placed in the top chamber of Matrigel-coated inserts (Corning, 354480), with medium containing 20% FBS added to the lower chamber. Cells were fixed and stained after 48 hours as described above. For both assays, the number of cells in five random fields was counted using an inverted microscope.

### In vivo metastasis assays

For the assessment of lung metastasis via tail vein injection, female BALB/c nude mice (aged 4–5 weeks) and NSG mice (aged 5–6 weeks) were obtained from SPF Biotechnology Company (Beijing). To analyze metastasis, 5 × 10^6^ viable HCT116 or SW480 cells were resuspended in 0.2 mL of PBS and administered into BALB/c or NSG mice through the lateral tail vein. The survival of the mice was monitored daily. Following a period of 42 days for HCT116 or 90 days for SW480, mice were euthanized. The lungs, livers, and other metastatic sites were extracted, photographed, and fixed in 4% paraformaldehyde (PFA). The tissues were then embedded in paraffin, sectioned, and stained with hematoxylin and eosin (H&E). The foci of visible lung and liver metastases were measured and counted under a microscope. Additionally, HCT116 cells tagged with luciferase were constructed. A total of 1 × 10^6^ cells resuspended in 0.2 mL of PBS were injected into the lateral tail vein of nude mice. After 21 days, mice were administered D-luciferin (150 mg/kg) intraperitoneally and imaged 15 minutes post-injection using an IVIS 100 Imaging System, with an acquisition time of 1 minute.

A mouse model of liver metastases was established through intrasplenic injection of HCT116 and SW480 cells. Briefly, the spleens of the mice were exposed via an incision on the left upper abdomen. For each mouse, 2 × 10^6^ HCT116 cells or 5 × 10^6^ SW480 cells were injected into the spleen in 50 μL of PBS, allowing the cells to be delivered to the liver through the portal circulation. Ten minutes post-injection, the spleen was removed and the abdominal cavity was closed. Thirty-two days after implantation, all mice were euthanized, and the number of tumor foci on the liver surface was counted. Liver tissues were fixed and stained with H&E to detect liver metastases.

### Nuclear/Cytosol fractionation

Fractionation of cellular nuclear and cytoplasmic proteins was conducted utilizing the NE-PER Nuclear and Cytoplasmic Extraction Reagents (Thermo Fisher Scientific, 78833), adhering to the manufacturer’s guidelines. In summary, cells were collected and resuspended in CER I, followed by incubation on ice for 10 minutes. Subsequently, CER II was introduced to the lysates, which were then vortexed and maintained on ice for an additional 10 minutes. The cell lysates underwent centrifugation for 5 minutes at 16,000 *g*, and the supernatant was retained as the cytoplasmic extract. The insoluble pellets were resuspended in ice-cold NER and vortexed at intervals of 10 minutes over a total duration of 40 minutes. The lysates were centrifuged at 4 °C for 10 minutes at 16,000 *g*, and the supernatant was collected as the nuclear fraction. The cytoplasmic and nuclear extracts were subjected to western blotting analysis. GAPDH and H3 served as loading controls.

### SiRNA transfection

HCT116 or SW480 cells were plated in 6-well plates at a density of 3 × 10^5^ cells per well. Following an incubation period of 16 to 18 hours, the pooled siRNAs targeting TRPS1 and ZEB1 were transfected into the cells using Lipofectamine RNAiMAX (Invitrogen, 13778150, CA, USA), with a final siRNA concentration of 50 nM. Simultaneously, transfection with corresponding nonsense negative controls (NC) was performed. After 48 hours, RNA and protein levels were assessed to quantify the downregulation of gene expression. All siRNAs were synthesized by Shanghai GenePharma Co., Ltd. The siRNA sequences utilized are provided in Supplementary Table 7.

### RNA isolation and quantitative real -time PCR (qRT-PCR)

RNA extraction from the cells was carried out using the RNA simple Total RNA Kit (TIANGEN, 4992858), adhering to the manufacturer’s protocol. Subsequently, a total of 1 μg of RNA was reverse transcribed into cDNA using the SMART Scribe Reverse Transcriptase (TaKaRa, 639536, Tokyo, Japan). To detect differential expression of ZEB1, SPARC, CDH1, and EpCAM in the cDNAs, qPCR was employed with TB Green® Fast qPCR Mix (TaKaRa, RR430). The amplification process was conducted on a 7500 Fast Real-time PCR System (Applied Biosystems, Waltham, MA, USA). GAPDH served as an internal control for normalizing RNA input. The relative expression levels of the target genes, defined as the fold change, were calculated in comparison to the gene expression of control groups using the delta delta Ct (ΔΔCt) formula. The primer sequences utilized are provided in Supplementary Table 7.

### RNA sequencing (RNA-seq) and data analysis

RNA extraction from HCT116 and SW480 cells was carried out using TRIzol reagent (Invitrogen, 15596018). The preparation of RNA-seq libraries was performed using the TruSeq Ribozero Kit (Illumina, RS-122-2103). These libraries were then sequenced on the Illumina NovaSeq platform. The raw RNA-seq reads were trimmed using Skewer (version 0.2.2) to eliminate adapter sequences and subsequently aligned to the human hg19 reference genome using STAR (version 2.4.2a). RSEM (version 1.2.29) was employed to quantify expression abundance. Differentially expressed genes (DEGs) were identified based on a |fold change| of >=2 and an adjusted *P*-value of <0.05, using edgeR. Functional enrichment analysis of genes differentially enriched in experimental groups was conducted using the Metascape database (accessible at https://metascape.org/gp/index.html). The data have been deposited in the Gene Expression Omnibus database under accession number GSE206729.

### CUT & Tag assay

The CUT & Tag assay was carried out utilizing the NovoNGS® CUT & Tag 3.0 High-Sensitivity Kit (NOVOPROTEIN, N259-YH01-01A, Suzhou, China), following the manufacturer’s protocols. Briefly, cells were incubated with activated NovoNGS ConA Beads. Subsequently, the bead-bound cells were permeabilized and incubated initially with Flag primary antibody (1:50, Cell Signaling, 14793), followed by incubation with anti-rabbit antibody (10 µg/mL; Abcam, ab6702, Cambridge, MA, USA). The diluted pAG-Tn5 adapter complex was then added, followed by the tagmentation reaction. The extracted DNA fragments were used for library preparation. The libraries were sequenced by Novogene on the Illumina NovaSeq 6000 platform. The raw DNA sequencing reads were preprocessed to filter out sequencing adapters, short-fragment reads, and other low-quality reads. Bowtie (version 0.12.8) was employed to map the cleaned reads to the human hg19 reference genome. Peak detection was conducted using MACS with a minimum *P*-value cut-off of 0.00001 (version 1.4.2, available at https://pypi.python.org/pypi/MACS/1.4.2). The peak recognition, overlap, subtraction, merge, and feature annotation analysis of enriched regions were performed using the Hypergeometric Optimization of Motif EnRichment 2 (HOMER2) suite (version 3.0, accessible at http://homer.ucsd.edu/homer/). Motif-density histograms were generated using HOMER for target regions and promoters, which were defined as −2 kb to +1 kb relative to the transcription start site (TSS). The data were deposited in the Gene Expression Omnibus database under accession number GSE206564.

### Chromatin immunoprecipitation (ChIP) and ChIP-qPCR

Chromatin immunoprecipitation (ChIP) assays were conducted utilizing the Pierce Agarose ChIP Kit (Pierce/Thermo Fisher Scientific, 26156), adhering to the provided instructions. Briefly, HCT116 and SW480 cells were subjected to crosslinking with 1% formaldehyde (Sigma, F8775, St. Louis, MO, USA) for a duration of 10 minutes, following which the reaction was terminated with 0.125 M glycine. The cell pellets were lysed using micrococcal nuclease on ice for 10 minutes. Subsequently, rabbit anti-Flag antibody (5 μg per sample) or isotype IgG control (5 μg per sample) was added to 5 μg of sonicated chromatin and incubated at 4 °C overnight. The complexes were then captured on Protein A/G magnetic beads. The immunoprecipitates were washed and subjected to de-crosslinking. The eluted chromatin was purified and utilized as a template in qPCR, which was performed on a 7500 Fast Real-time PCR System (Applied Biosystems, USA). The primers employed for ChIP assays are enumerated in Supplementary Table 7.

### Dual-luciferase reporter assay

The promoter sequence of ZEB1, specifically the fragment spanning −2079 to +200, along with its three truncated derivatives (fragment 1: −2079 to ion status was wild type in one single cell 1079; fragment 2: −1080 to +200; and fragment 3: −200 to +200), were cloned into the pGL3-Basic plasmid. Subsequently, 293 T cells were seeded at a density of 2.5 × 10^5^ cells per well into 12-well plates and cultured until they reached approximately 70% confluence. The cells were then co-transfected with 200 ng/well of the pGL3-Basic plasmid harboring the specific promoter sequence, 500 ng/well of the PLBMV-TRPS1 WT/MT plasmid, and 10 ng/well of the pRL-TK plasmid. After 48 hours, the cells were assayed for fluorescence intensity utilizing the Dual-Luciferase Reporter Assay System (Promega, E1910, Madison, WI). Prior to luminescence detection, the cells were washed with PBS and lysed using 250 μL of PLB per well, incubated for 15 minutes at room temperature. A 20 μL aliquot of the lysate was transferred to a 96-well plate (Corning, 3917, Corning, NY) for luminescence measurement. The outcomes are presented as the relative luciferase activity, calculated as the ratio of firefly luciferase intensity to renilla luciferase intensity.

### Immunohistochemistry (IHC) analysis

Formalin-fixed, paraffin-embedded tissue sections underwent deparaffinization and rehydration through a graded series of ethanol solutions, followed by immersion in PBS buffer. Antigen retrieval was achieved by heating the slides for 10 minutes at 97 °C in sodium citrate buffer, subsequently followed by incubation in 0.3% H_2_O_2_ for 10 minutes to inhibit endogenous peroxidase activity. To block nonspecific binding sites, the sections were incubated with 10% normal goat serum for 30 minutes. Overnight incubation at 4 °C was performed with the primary antibody (rabbit anti-ZEB1, diluted 1:100, Cell Signaling, 3396). Detection was carried out using the LSAB+ System-HRP (Dako/Agilent, Santa Clara, CA, USA). The staining intensity was categorized as 0 (negative), 1 (weak), 2 (medium), or 3 (strong). H scores were determined by multiplying the staining intensity by the percentage of positive cells, yielding a range of overall scores from 0 to 300. The histological specimens were evaluated by a trained pathologist with expertise in CRC disease.

### Statistical analysis

Data are presented as the mean ± SEM. For unpaired group comparisons, the two-tailed unpaired Student’s *t*-test was utilized. Multiple comparisons were analyzed using one-way ANOVA. Contingency tables were assessed with the Fisher’s exact test. The comparison of Kaplan-Meier overall survival curves was conducted using the log-rank (Mantel-Cox) test. Statistical significance was set at *P*-values < 0.05. Significant differences are denoted as follows: * for *P* < 0.05, ** for *P* < 0.01, *** for *P* < 0.001, and **** for *P* < 0.0001. All statistical analyses were performed using GraphPad Prism 8.0 (San Diego, CA, USA) or SPSS, version 19.0 software (released in 2010 by IBM Corp., Armonk, NY, USA).

## Supplementary information

Supplementary Materials

Supplementary Table 1

Supplementary Table 2

Supplementary Table 3

Supplementary Table 4

Supplementary Table 5

Supplementary Table 6

Supplementary Table 7

Supplementary Table 8

Supplementary Table 9

## Data Availability

Data have been submitted to the Gene Expression Omnibus database (accession numbers GSE206729 and GSE206564).
