# Supplementary material for: Single-cell exome sequencing reveals polyclonal seeding and TRPS1 mutations in colon cancer metastasis
Source: Signal Transduct Target Ther. 2024 Sep 23;9:247. doi: 10.1038/s41392-024-01960-8 (PMC11417107; doi:10.1038/s41392-024-01960-8)
Supplement: Supplementary file 1 — Supplementary Materials [file 41392_2024_1960_MOESM1_ESM.docx]

Supplementary Materials for

Single-cell exome sequencing reveals polyclonal seeding and Transcriptional Repressor GATA Binding 1 (TRPS1) mutations in colon cancer metastasis

Jianqiang Cai*, Weilong Zhang*, Yalan Lu*, Wenjie Liu*, Haitao Zhou*, Mei Liu, Xinyu Bi, Jianmei Liu, Jinghua Chen, Yanjiang Yin, Yiqiao Deng, Zhiwen Luo, Yi Yang, Qichen Chen, Xiao Chen, Zheng Xu, Yueyang Zhang, Chaoling Wu, Qizhao Long, Chunyuan Huang, Changjian Yan, Yan Liu, Lei Guo, Weihua Li, Pei Yuan, Yucheng Jiao, Wei Song, Xiaobing Wang, Zhen Huang^+^, Jianming Ying^+^, Hong Zhao^+^

Correspondence to: [zhaohong@cicams.ac.cn](mailto:zhaohong@cicams.ac.cn), [jmying@cicams.ac.cn](mailto:jmying@cicams.ac.cn), zhen.huang@cicams.ac.cn

This PDF file includes:

Figures. S1 to S17

Other Supplementary Materials for this manuscript include the following:

Table S1 to S9

SUPPLEMENTARY FIGURES

Supplementary Figure 1. Clean data production of single-cell exome sequencing.

The coverage and depth of single-cell exome sequencing data from each cell.

Supplementary Figure 2. Boxplots for (a) false positive discovery rate and (b) allele dropout rates in single-cell exome sequencing.


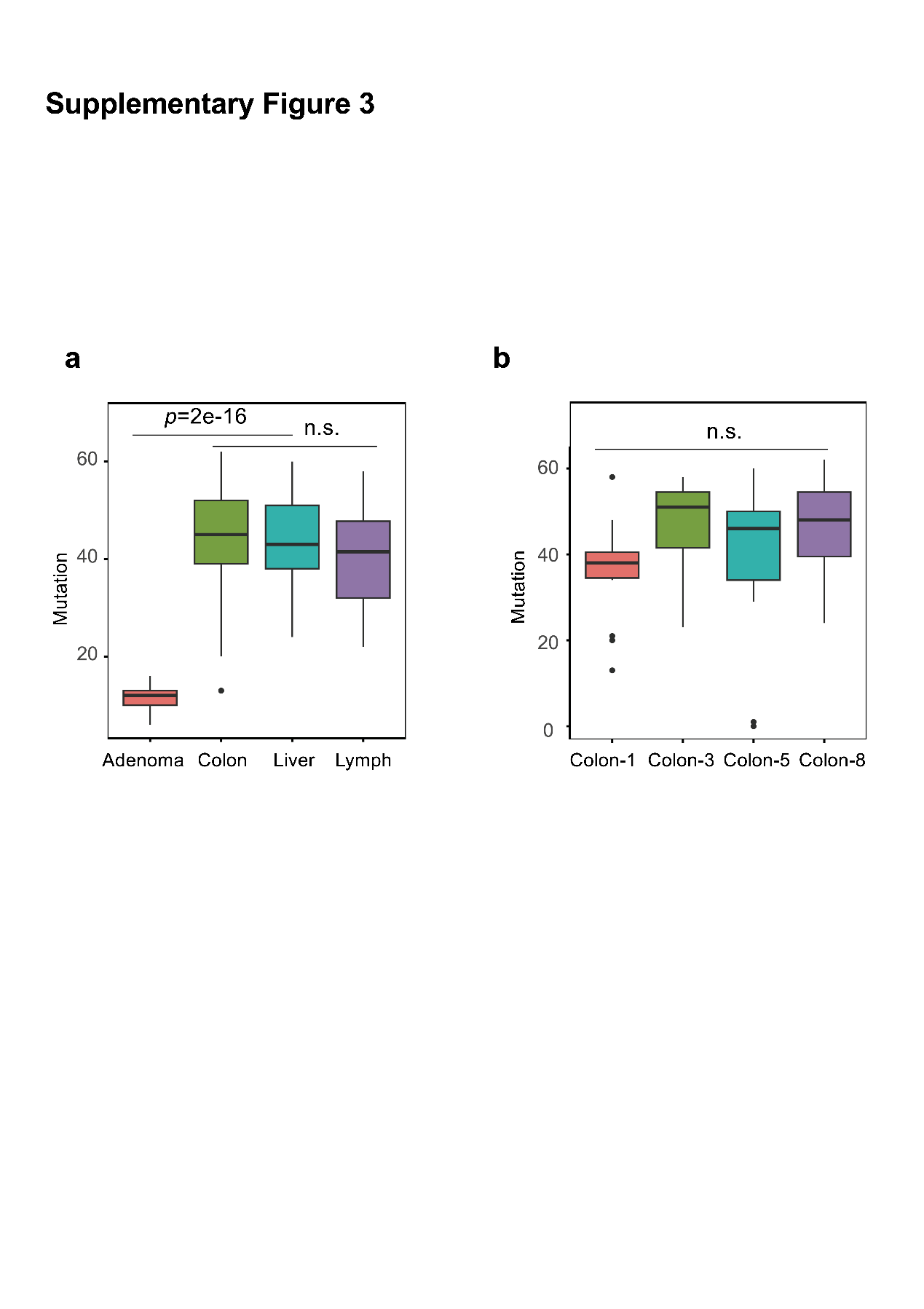


Supplementary Figure 3. The mutation counts in different cancer tissues.

(a) Mutation counts of the different tumors, including primary, adenoma, liver and lymphatic metastases representing inter-tumor differences. (b) Mutation counts of different areas from the primary tumor representing intra-tumor differences.

Supplementary Figure 4. The mutation counts in different cancer tissues.

(a) Diagram showing sites of specimens harvested from adenoma, primary colon cancer and liver and lymphatic metastases from a single stage IV colon cancer patient. (b) The overlap of mutations in primary colon, liver and lymph tumors is 41.9% (49/117), and in adenoma, colon, liver and lymph tumors, 0.85% (1/117). (c) SNVs specific appear to each tumor type/process in colon cancer evolution. All SNVs shown are protein damaging SNVs. Mutations associated with tumors are indicated by the colors in the legend.

Supplementary Figure 5. Schematic representation of liver and lymphatic metastases originating from tumor cells in a region between colon 1 and colon 5.


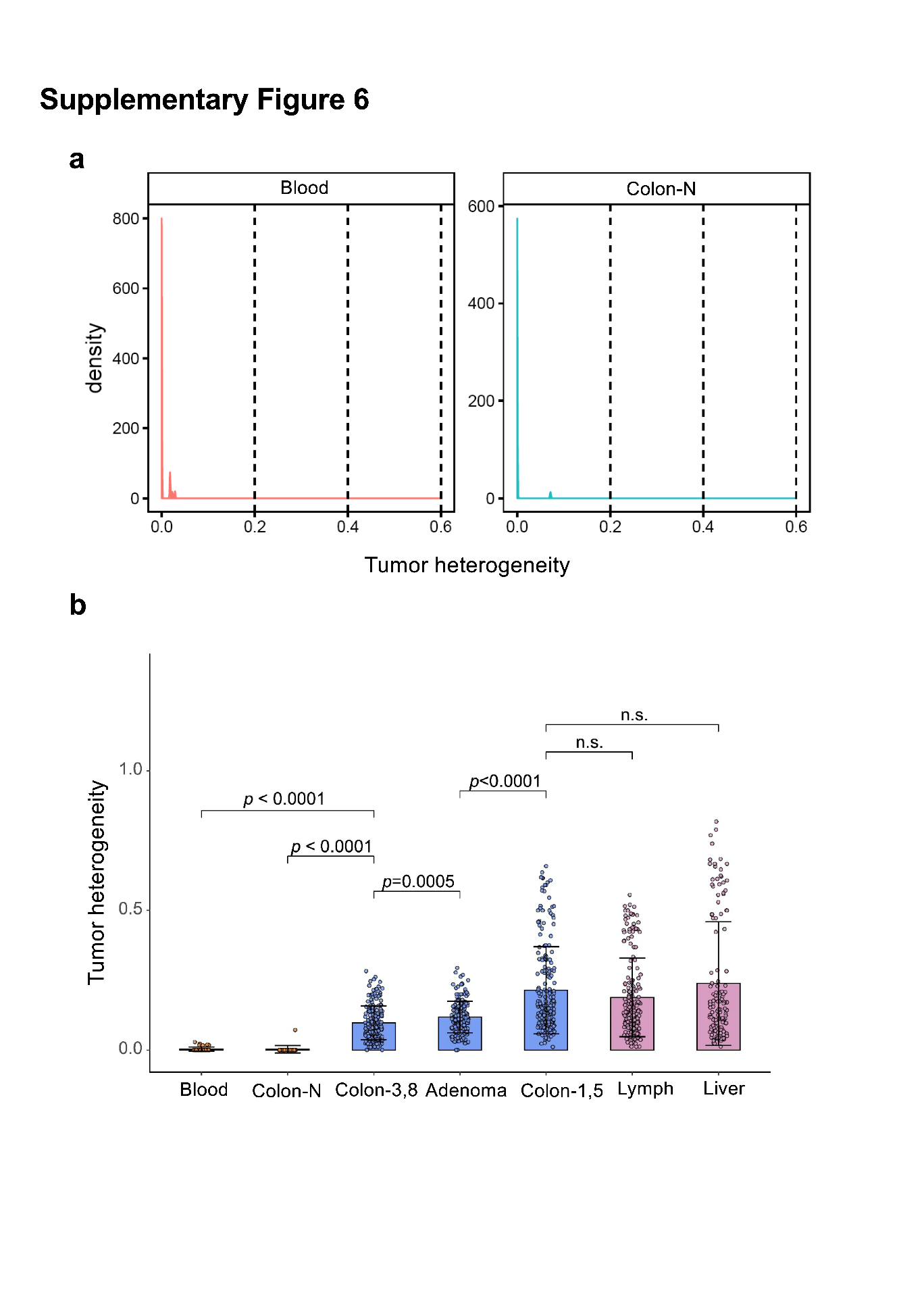


Supplementary Figure 6. Intra-tissue heterogeneity of the blood and normal colon tissues.

(a) Density curve showing the distribution of intra-tissue heterogeneity for the blood and normal colon tissues. (b) Bar plot showing heterogeneity of the blood, normal colon tissue, and primary and metastatic tumor tissues. Unpaired t test, two-sided.

Supplementary Figure 7. Mutational signature analysis of 150 single cells and 7 related tumor tissues genomes using the Wellcome Trust Sanger Institute mutational signatures framework.

Two mutational signature patterns (signature A, signature B) observed in the 150 single cell genomes from the Wellcome Trust Sanger Institute mutational signatures framework. The fractions represent the proportion of each base combination in the 96 base combinations.


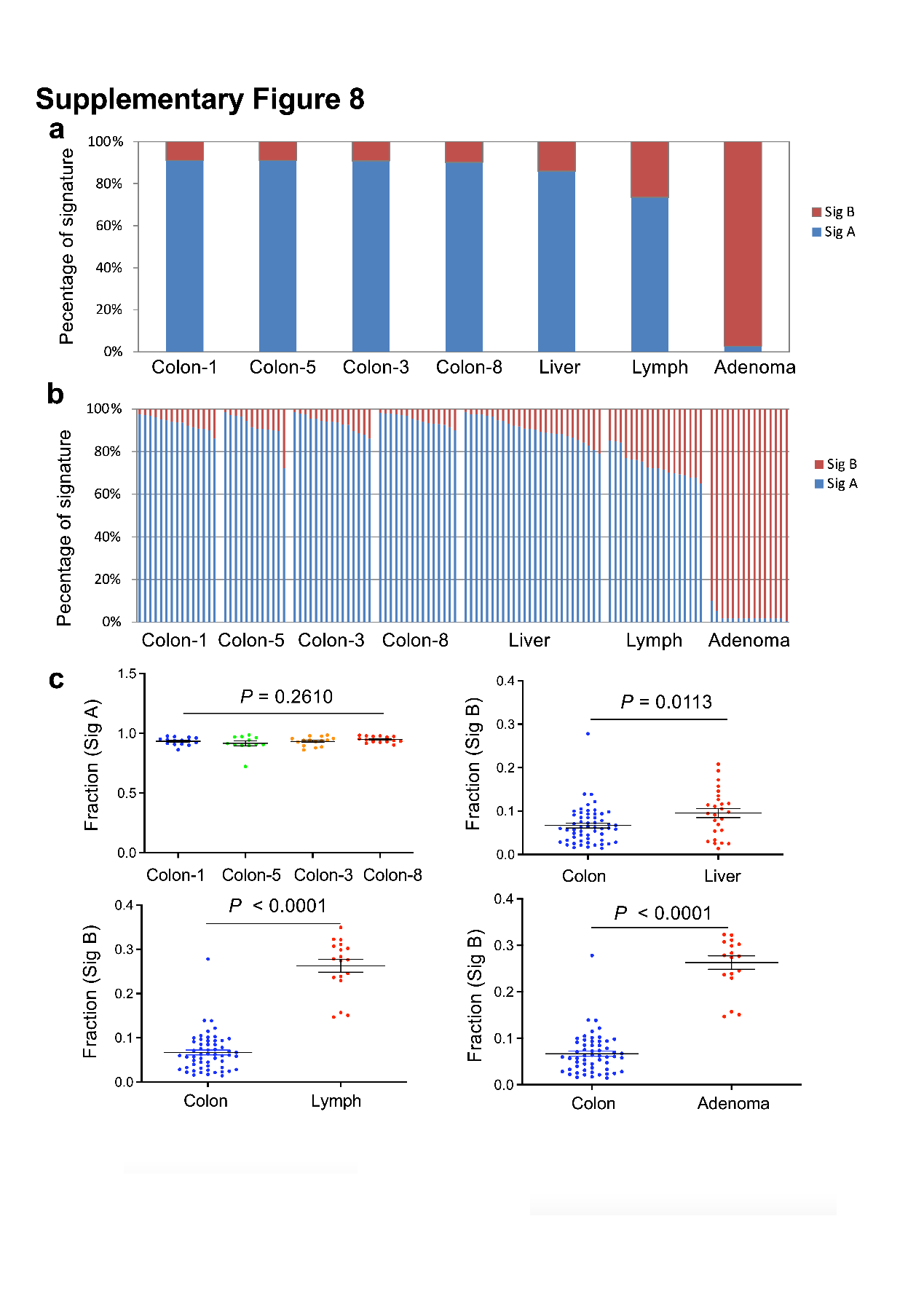


Supplementary Figure 8. Mutational signature analysis of 150 single cells and 7 related tumor tissues genomes using the Wellcome Trust Sanger Institute mutational signatures framework.

(a) The proportion of mutational A and B signatures observed in the 7 tumor tissues.

(b) The proportion of mutational A and B signatures observed in the 150 single cells.

(c) Comparison of the proportion of mutational A and B signatures in each cell from primary (individual areas and together) and metastatic tissues. Unpaired t test, two-sided.

Supplementary Figure 9. Related to Figure 4. Single-cell RNA-seq analysis of primary CRC and matched liver metastases samples.

(a) UMAP plot showing the cancer cells of liver metastases from each patient. Colors indicate different patients.

(b) UMAP plot showing the cancer cells of liver metastases were separated into 5 subgroups. Colors indicate different subgroups.

(c) Box diagram showing the CNV scores for different clusters. *** means P < 0.001.

(d-i) Sankey plots showing the association subgroups of cancer cells in primary CRC and matched liver metastases in each patient.

Supplementary figure 10. Related to Figure 4. Enrichment analysis of expressed differential genes among subgroups with different metastasis contribution degrees.

Bar plot showing results of GO analysis applied to differentially expressed genes of subgroups of primary CRC (cluster 2, 3 vs. other clusters).

Supplementary Figure 11. Correlation between EMT score and clinical information in a validated single-cell transcriptome cohort which included six fresh tumor samples from liver metastases.

(a) Analysis comparing EMT scores between patients presenting with lymphatic metastasis versus those without.

(b) Analysis comparing EMT scores between patients responding positively to preoperative chemotherapy and those who did not respond.

(c) Analysis comparing EMT scores between patients with comorbidities and those without.

(d) Analysis comparing EMT scores between patients exhibiting microvascular invasion (MVI) and those without.

(e) Comparative analysis of EMT scores between patients in T4 stage versus those in T3 stage. All P-values were analyzed using Wilcox rank-sum test.


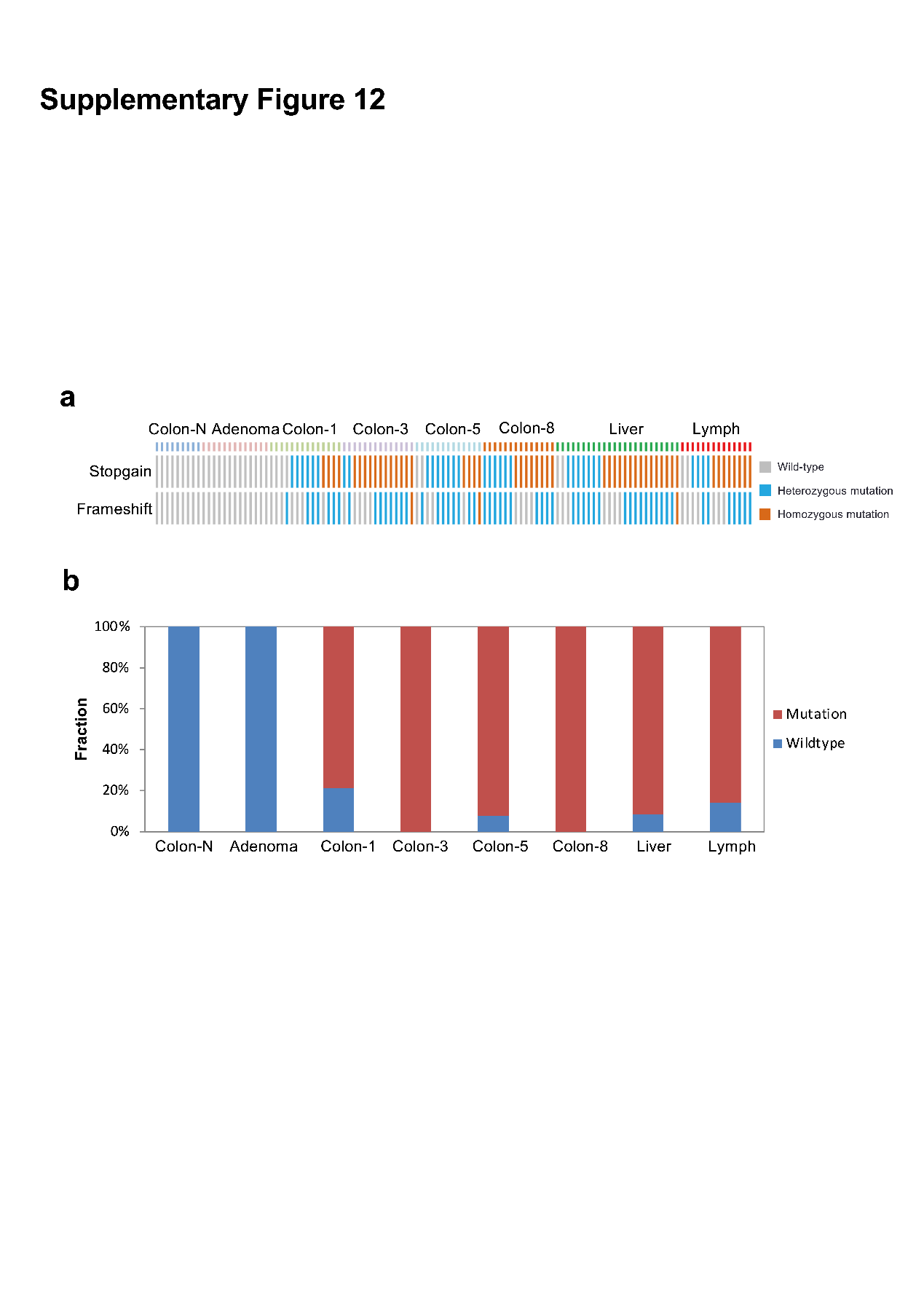


Supplementary Figure 12. *APC* gene mutation status of single cells.

(a) *APC* gene mutation status of single cells for each tissue.

(b) Proportion of mutations identified in each tissue.

Supplementary Figure 13. Double hit model derived from single cells.

Schematic representation of a possible double hit model based on the pattern of *APC* gene mutations derived from single cells.

Supplementary Figure 14. IGV showing the mutation of *APC* gene.

(a) IGV showing the stopgain (upper panel) *APC* gene mutations in the colon-8 tissue from bulk whole exome sequencing.

(b) IGV showing the frameshift (lower panel) *APC* gene mutations in the colon-8 tissue from bulk whole exome sequencing.


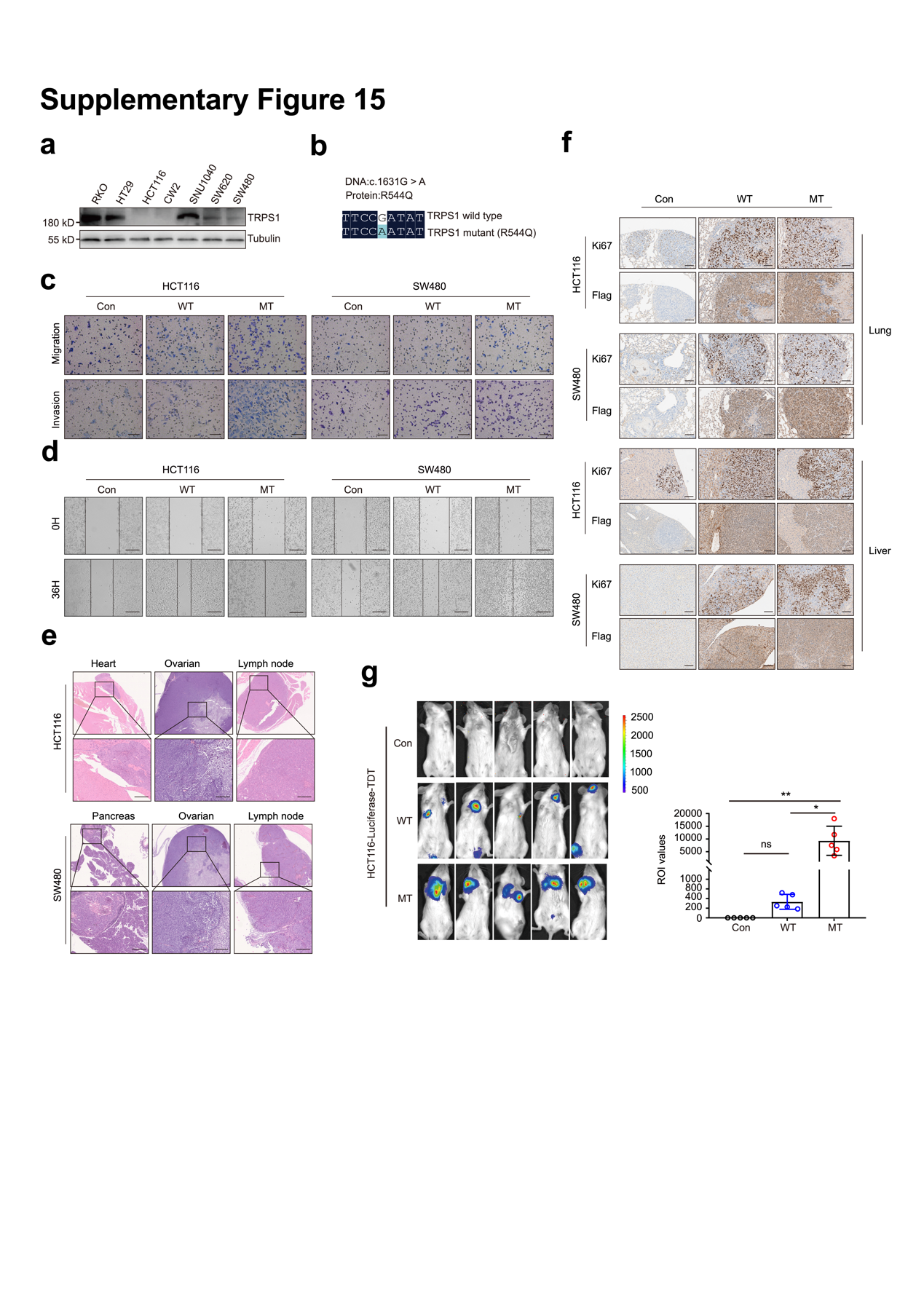


Supplementary Figure 15. Related to Figure 5. TRPS1 R544Q mutation CRC cell lines construction and metastasis phenotypic analysis *in vitro* and *in vivo*.

(a) Western blot analysis to measure the expression of TRPS1 in CRC cell lines.

(b) DNA sequencing to confirm the *TRPS1* R544Q mutant in HCT116 and SW480 cells.

(c) Representative images of migration and invasion assays for the indicated colorectal cancer cells related to Figure 6b-c.

(d) Images of scratch wound assays at 36 h for conﬂuent HCT116 and SW480 cells with ectopic expression of Con, WT and MT, related to Figure 6 d. Scale bars, 100 μm. (e) Representative H&E staining of heart, ovarian and lymph node from mice injected with HCT116 cells (top) and pancreas, ovarian and lymph node from mice injected with SW480 cells (bottom). Scale bars, 125 μm.

(f) Respective images of immuno-histochemistry for Flag and Ki67 from HCT116 and SW480 groups, related to Figure 6G. Scale bars, 100 μm.

(g) Bioluminescence images (left) and quantification of luciferase activity (right) of five anesthetized mice from each group by IVIS. All data are represented as the mean ± SEM, ns, not significant, *, *P* < 0.05, **, *P* < 0.01.


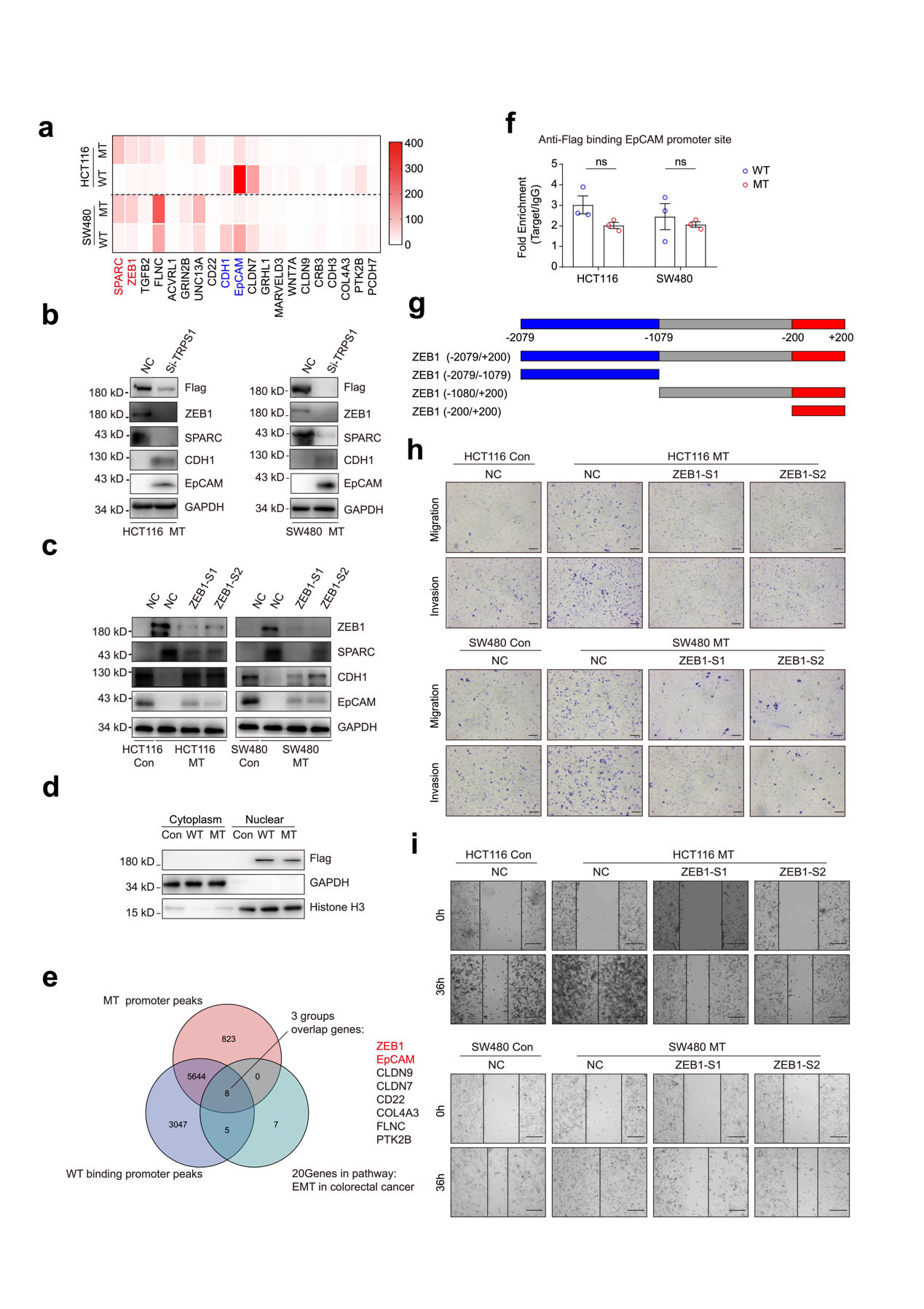


Supplementary Figure 16. Related to Figure 6. The mechanism and phenotypic analysis of *TRPS1* mutant (R544Q) promotes the colorectal cancer cell metastases.

(a) The expression of 20 DEGs associated with EMT in HCT116 and SW480 WT versus MT cells.

(b) Western blot analyses to detect protein levels of ZEB1, SPARC, CDH1 and EpCAM in indicated CRC cells with TRPS1 knockdown.

(c) Western blot analyses to detect protein levels of SPARC, CDH1 and EpCAM in HCT16 (left) and SW480 (right) cells with ZEB1 knockdown.

(d) Western blot analyses of cytoplasmic and nuclear fractions from indicated HCT116 cells. GAPDH and Histone H3 were used as cytoplasmic and nuclear control markers, respectively.

(e) Venn diagram showing the overlap among EMT gene set in RNA-seq and enriched by Flag CUT&Tag-Seq in HCT116 WT and MT cells.

(f) ChIP-qPCR analysis of TRPS1 enrichment at the *EpCAM* promoter region in *TRPS1* WT and MT (R544Q) cells.

(g) Schematic representation of *ZEB1* promoter truncation constructs.

(h) Representative images of migration and invasion assays of the indicated colorectal cancer cell lines related to Figure 7 (g-h).

(i) Images of scratch wound assays at 36 h for HCT116 and SW480 cells with ZEB1 knockdown in the rescue assays related to Figure7i. Scale bars, 100 μm. All data are represented as the mean ± SEM, ns, not significant.


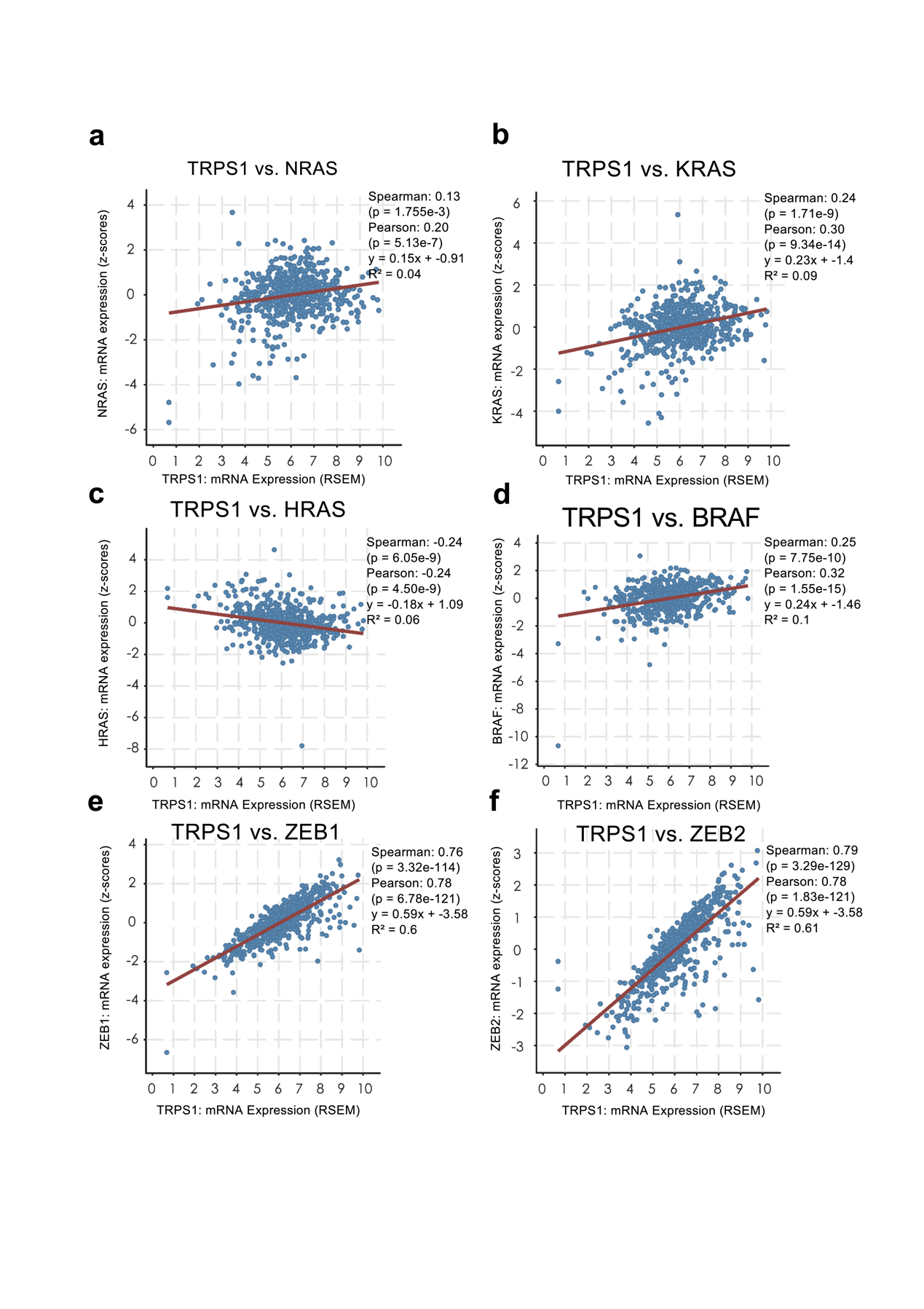


Supplementary Figure 17. The relationship between TRPS1 expression levels and the expression of NRAS, KRAS, HRAS, BRAF, ZEB1, and ZEB2.
